# Immunogenicity profiling of protein antigens from capsular group B *Neisseria meningitidis*

**DOI:** 10.1038/s41598-019-43139-0

**Published:** 2019-05-02

**Authors:** Amaka M. Awanye, Chun-Mien Chang, Jun X. Wheeler, Hannah Chan, Leanne Marsay, Christina Dold, Christine S. Rollier, Louise E. Bird, Joanne E. Nettleship, Raymond J. Owens, Andrew J. Pollard, Jeremy P. Derrick

**Affiliations:** 1 Lydia Becker Institute of Immunology and Inflammation, School of Biological Sciences, Faculty of Biology, Medicine and Health, Manchester Academic Health Science Centre, University of Manchester, Manchester, M13 9PL UK; 2 National Institute for Biological Standards and Control, Blanche Lane, South Mimms, Hertfordshire, EN6 3QG UK; 3 Oxford Vaccine Group, Department of Paediatrics, University of Oxford, and the NIHR Oxford Biomedical Research Centre, Oxford, OX3 7LE UK; 4 Oxford Protein Production Facility, Research Complex at Harwell, Rutherford Appleton Laboratory, Harwell, Didcot, OX11 0FA UK

**Keywords:** Lipoproteins, Protein vaccines, Bacteriology, Protein vaccines

## Abstract

Outer membrane vesicle (OMV)- based vaccines have been used to provide strain-specific protection against capsular group B *Neisseria meningitidis* infections, but the full breadth of the immune response against the components of the OMV has not been established. Sera from adults vaccinated with an OMV vaccine were used to screen 91 outer membrane proteins (OMPs) incorporated in an antigen microarray panel. Antigen-specific IgG levels were quantified pre-vaccination, and after 12 and 18 weeks. These results were compared with IgG levels from mice vaccinated with the same OMV vaccine. The repertoires of highly responding antigens in humans and mice overlapped, but were not identical. The highest responding antigens to human IgG comprised four integral OMPs (PorA, PorB, OpcA and PilQ), a protein which promotes the stability of PorA and PorB (RmpM) and two lipoproteins (BamC and GNA1162). These observations will assist in evaluating the role of minor antigen components within OMVs in providing protection against meningococcal infection. In addition, the relative dominance of responses to integral OMPs in humans emphasizes the importance of this subclass and points to the value of maintaining conformational epitopes from integral membrane proteins in vaccine formulations.

## Introduction

*Neisseria meningitidis* is the causative pathogen of meningococcal meningitis and septicaemia. More than 90% of the cases of invasive disease worldwide are caused by six capsular groups, A, B, C, W, X, and Y^1^. Vaccines based on capsular polysaccharide (CPS) have been effective in preventing and controlling outbreaks of disease attributed to capsular groups A, C, Y and W for many years, and several pentavalent X-polysaccharide-containing vaccines are in development in India. Unfortunately, although capsular group B *N. meningitidis* (NmB) organisms contribute to most disease cases recorded in developed countries^2^, there are safety concerns about the use of capsular group B CPS, as it shares similarities to polysialic acid-containing structures in human foetal brain tissue^3,4^. Outer membrane vesicles (OMVs), which are derived from meningococcal growth cultures by detergent extraction, have been used successfully as vaccines to control clonal outbreaks of NmB infection^5–7^. However, the sequence hypervariability of several NmB OMPs has hampered the development of a universal vaccine using a single OMV. The protection induced by OMV vaccines is largely directed at the hypervariable PorA antigen, the most abundant protein in OMVs^8^. There is therefore a need to identify other vaccine candidates that are more conserved and can also provide protection against NmB infections. Proteomic analyses of OMVs have identified well over 100 different protein components, including a large number of integral outer membrane proteins (iOMPs) which, for the purposes of this paper, we define as proteins where the polypeptide chain completely spans the outer membrane at least once^9,10^. iOMPs generally form membrane-spanning β-barrel folds and several crystal structures are known for meningococcal proteins^11–14^.

The implementation of reverse vaccinology (RV) has driven the development of 4C-MenB (tradename Bexsero®), a vaccine which comprises recombinant protein antigens in combination with an OMV component^15^. RV is a multistep process, starting with bioinformatic prediction of surface-expressed proteins, followed by their individual expression and evaluation of immunogenicity in a suitable vaccination model^16^. However, iOMPs were excluded during the selection process, presumably due to challenges associated with protein expression and folding into their native three-dimensional structures^17,18^. Clinical trials with the 4C-MenB vaccine showed that inclusion of OMVs from a single strain improved the protection conferred by the recombinant antigens (NadA, NHBA, fHbp, GNA2091 and GNA1030)^19–23^. These observations point to the potential importance of other OMPs, including iOMPs, in helping to confer protection.

Protein microarrays provide a high throughput platform for the identification of protein-protein interactions^24^. Screening using antisera derived from vaccinees can be used to identify potential vaccine antigens, as well as diagnostic markers^25,26^. In order to carry out such a screen, a complete library of component antigens is ideally required. It is, however, very difficult in practical terms to obtain a comprehensive library of proteins, due to the challenges associated with protein expression and ensuring correct protein folding. Some researchers have used high throughput *in vitro* transcription and translation (IVTT) methods, where proteins are produced *in situ*, in a cell-free system. This approach is simple, fast and convenient for expressing toxic proteins: it has been used in the profiling of immune responses to infection and identification of diagnostic markers, for example^25–28^. However, IVTT has some drawbacks: first, there is a need to standardize the amount and homogeneity of the protein in each sample, which can be difficult. Second, many longer polypeptide chains do not express well and formation of multimers is difficult to verify. Finally, proteins need to be folded into their native conformation to preserve conformational epitopes and, again, this can be difficult to verify directly.

In previous work Steller *et al*. adopted a different approach, by individually expressing and purifying 67 out of 102 phase-variable NmB antigens in recombinant form and using them to create a microarray which was then screened against antisera obtained from meningitis and sepsis patients^29^. However, purification of iOMPs was carried out under denaturing conditions and hence the conformational epitopes from these proteins were lost: for example, only one out of 20 patient sera tested was reactive against PorA. Here we designed a panel of 91 OMPs, including iOMPs, which have been identified as potential vaccine candidates and are also OMV components^10,15^. The antisera used were derived from a Phase I study using an OMV preparation from a modified NmB strain H44/76, termed MenPF-1, in which the *fetA* promoter was modified to ensure constitutive expression of the iron transporter FetA^8,30,31^. This work represents the most comprehensive attempt yet to compare the immunogenicity of OMP components of an *N. meningitidis* OMV vaccine in humans and mice. The results highlight the shortcomings of murine antibody responses as predictors of the human immune response and have value in helping to refine the components of meningococcal vaccines in development.

## Results

### Design and manufacture of the meningococcal antigen microarray

Information was used from previous proteomics and FACS analysis to identify OMV component proteins as potential targets for inclusion in the microarray^10,15,32,33^. In addition, other protein antigens were considered which, on the basis of more recent work, justified their inclusion (eg Adhesin Complex Protein)^34^. The sequences of individual open reading frames (ORFs) were searched against the Protein Data Bank, to extract information from homologs about domain structure and optimal expression strategies (eg selection of N- and C-termini to allow isolation of specific domains if expression of the entire protein was likely to prove challenging). The selected antigens covered a diverse range of biological functions, including adhesion, transport and immuno-modulation (Table 1). Not all proteins were necessarily predicted to be surface-exposed; some periplasmic proteins were included because they were present in OMV preparations and potentially could elicit an immunogenic response. In addition, we carried out a separate proteomic analysis on the OMV preparation using multidimensional LC-MS/MS peptide sequencing and searching of the *N. meningitidis* serogroup B/serotype 15 (strain H44/76) FASTA database. A total of 557 proteins were identified which confirmed the presence of key OMPs in the OMV preparation, including those identified as major responding antigens (Supplementary Table S1).

**Table 1:** Classification of purified meningococcal OMP antigens based on their biological functions.

	Function^a^	Total	Soluble^b^	Refolded	Folded^c^	Unfolded
1	Adhesion	9	5	4	9	0
2	Redox homeostasis	6	6	0	5	1
3	Lysozyme inhibition	2	2	0	2	0
4	Peptidoglycan binding	2	2	0	2	0
5	OM assembly	6	2	4	6	0
6	Protein folding	7	7	0	7	0
7	Metabolism	3	3	0	2	1
8	Unknown function	4	2	2	3	1
9	Protein assembly	1	1	0	0	1
10	Peptidoglycan synthesis	4	3	1	4	0
11	Immunomodulation	4	1	3	4	0
12	OM stability	1	1	0	1	0
13	Macromolecular transport	17	5	12	14	3
14	Micromolecular transport	25	16	9	22	3
		**91**	**56**	**35**	**81**	**10**

^a^Functional classification of selected proteins was carried out based on entries in the Pfam database^75^.

^b^Proteins were classified as soluble or refolded based on their solubility after initial cell disruption.

^c^Based on thermal fluorescence denaturation profile.

**Figure 1:**
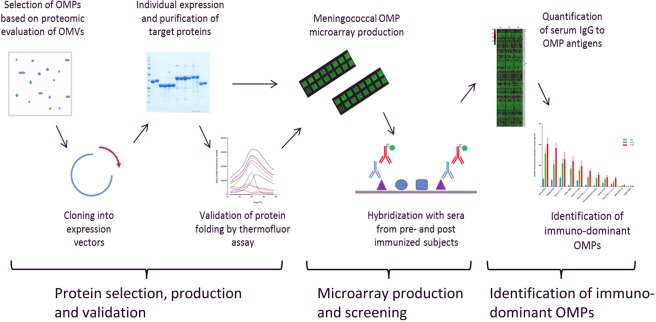
Work flow for identification of immuno-dominant OMPs. Our strategy is divided into three main steps, namely protein selection, production and validation of expression and folding; protein microarray production and screening and quantification of sero-reactivity and identification of immuno-dominant OMPs.

The overall strategy adopted for fabrication of the microarray is shown in Fig. 1. Soluble proteins were expressed and purified by metal chelate affinity chromatography, followed by size exclusion chromatography (SEC). Predicted iOMPs were expressed without their signal sequences, which led to formation of inclusion bodies: they were re-solubilized in urea and refolded by rapid dilution into buffer containing detergent before purification by the same processes. During purification by SEC, we observed that some proteins eluted in more than one oligomeric state (eg monomers and dimers); they were collected separately to determine the contribution of oligomerization to the immune response, bringing the total of separate antigens on the array to 91. More details on the individual proteins are given in Supplementary Table S2.

To evaluate the degree of folding of each purified antigen, we used the measurement of fluorescence of Sypro Orange dye, as a function of temperature: this is a widely used and convenient method which lends itself to high throughput applications^35^. An example is shown in Supplementary Fig. S1; a peak in the first derivative change in fluorescence with temperature is indicative of an unfolding event. In this example, Neisserial surface protein A (NspA), an iOMP, shows a transition corresponding to the melting temperature at approximately 60 °C, compared with the IgA-specific endopeptidase sample, which shows no transition feature. Of the 91 NmB OMPs in the array, 81 (~90%) gave evidence of folded structure by this criterion. Human and mouse IgG, and Epstein-Barr nuclear antigen 1 (EBNA-1) were included on the array as controls^36^. Interference arising from antibody binding to traces of contaminant proteins was eliminated by addition of an *E. coli* soluble lysate and a membrane–enriched extract during primary antibody hybridization^37^. Microarray glass slides coated with porous nitrocellulose (Grace Biolabs) were used to immobilize the proteins on the slide. The nitrocellulose membrane is porous and captures proteins within its fibrous matrix. This approach offers optimal binding capacity, retains protein conformation and provides longer shelf-life stability^38,39^. For refolded proteins such as iOMPs, which do not adhere optimally on glass slides, this approach was most suitable for immobilization. The proteins were printed using a non-contact, on-the-fly technology which offers more advantages over contact printing, such as high precision, excellent spot morphology and also avoids protein damage during the contact printing^40^. Preliminary screening with anti-histidine primary antibodies showed excellent reproducibility within replicate spots and between different miniarrays. All protein spots demonstrated a good morphology and consistency, with no merging.

### Identification of antigens providing strongest IgG reactivity in human and murine antisera after OMV vaccination

**Figure 2:**
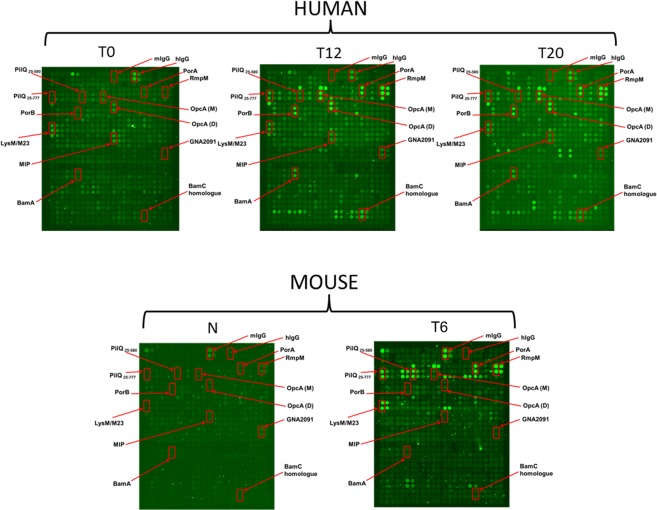
Exemplar raw data microarrays of human or mouse IgG seroreactivity. The microarrays were hybridized with human or mouse sera and developed for total IgG. T0 or naïve (N) represents pre-vaccination serum, T12 and T20 represent post-vaccination collection the 12 and 20 week time points respectively from the human high dose vaccinees while T6 represents terminal bleed from vaccinated mice after 2 doses. Spots representing highly immunogenic proteins in human or mouse sera are blocked in red. Duplicate spots are shown; the third replicate spot is not in close proximity to the first two spots.

Serum samples obtained from immunized mice or adult human participants of an OMV vaccine phase I trial were used to probe the microarray slides. The human antisera were obtained from a previous study: high (50 μg OMV protein) or low (25 μg OMV protein) dose vaccine was given every 8 weeks and serum samples collected 4 weeks after each vaccination^30^. Serum samples used in this study were collected at the following time-points: pre-vaccination (T0), post-second (T12) and post-third vaccinations (T20), 12 and 20 weeks post-vaccination respectively. The mouse antisera are from the terminal bleed of 10 BALB/c mice after two inoculations (3 weeks apart) with 2.5 μg OMV protein^8^. Protein spots were hybridized with sera from pre- and post-vaccination samples and bound IgG antibodies were detected by the fluorescence signal intensity. Given that all OMPs were printed at the same concentration and most (~88%) retained their native conformation, differences in signal intensity are attributable to the concentration of specific IgG antibodies in the serum. Examples of sample data for human and mouse sera are shown in Fig. 2- the increase in IgG reactivity following inoculation is readily apparent.

**Figure 3:**
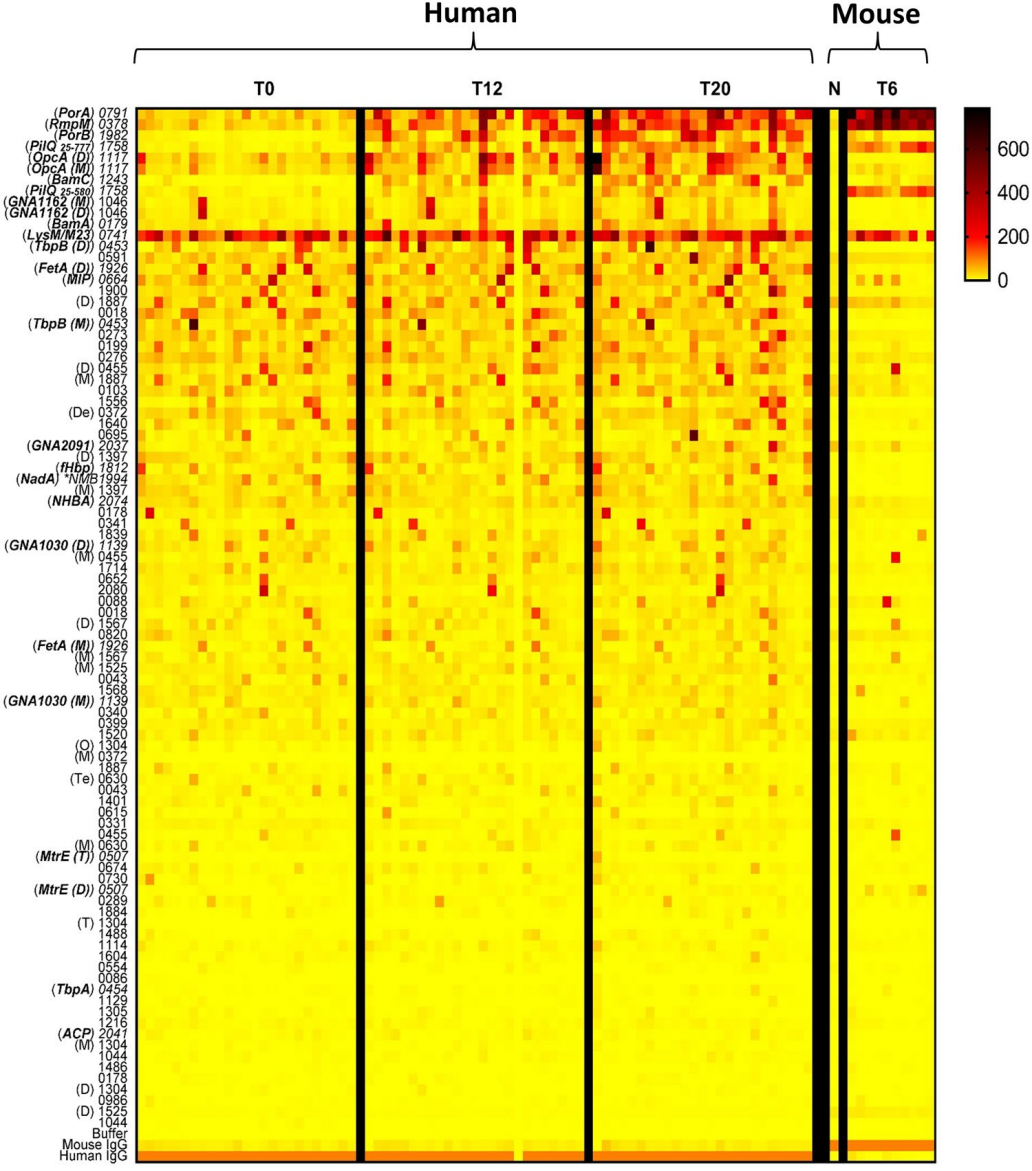
IgG seroreactivity of immuno-dominant OMPs at different vaccination time points. Reactivity of immunogenic proteins to pre- (T0 or N) and post-vaccination (T12 or T20) sera from 25 human vaccinees and 10 mice (T6) are quantified and represented as a heat map. The colour blocks represent the fluorescence signal intensity for reactivity against total IgG antibodies in serum. Antigens are ranked according to magnitude of difference between human T20 and T0. PilQ_25–777_ represents the full length protein while PilQ_25–580_ is missing the C-domain. Proteins are represented as the last 4 digits of their gene numbers (NMBH4476_), letters represent the oligomeric state (M, monomer; D, dimer; T, trimer; Te, tetramer; O, octamer; De, decamer). *NMB 1994 (NadA) is not expressed in strain H44/76 and was obtained from MC58 strain. More details on the individual proteins are given in Supplementary Table S2.

The serum IgG reactivity for both human high dose and mouse OMV vaccinations to individual purified protein antigens are summarised as a heat map in Fig. 3. IgG responses were highly variable between individuals, with some human vaccinees showing strong reaction against specific antigens at T0, as well as T12 and T20. Similar results were obtained for sera from the human low dose group, but were generally subject to more variation and are omitted here. Only a minority of antigens, at the top of the heat map, appeared to consistently stimulate a response at T12 and T20, relative to T0, in most human vaccine recipients. Understandably, the responses in mice were more consistent, with fewer instances of individual animals reacting against a particular antigen. The LysM/M23 domain protein maintained a consistently high reactivity before and after vaccination which could originate from non-specific cross-reactivity.

The major IgG reactive antigens for human and mouse antisera are summarised in Table 2. We identified seven different proteins for human and four for mouse which showed increases (p < 0.05) in IgG reactivity after vaccination. All identified highly sero-reactive OMPs retained folded characteristics, as judged by the Thermofluor assay (Supplementary Fig. S2). Of the seven antigens reactive against human antisera, four are iOMPs; of the remaining three, two are lipoproteins (GNA1162 and BamC) and the remaining antigen has a role in stabilising PorA/PorB trimers (RmpM^41^). Fewer antigens were identified from the mouse antisera, possibly because there were fewer individual serum samples and/or the mice are from an inbred strain; LysM/M23 is listed in Table 2 because it met the criteria for inclusion but, as noted above, it consistently showed a high background. Several antigens appear more than once in the list, either as a consequence of being in a different engineered form (PilQ) or oligomeric state (eg OpcA). Despite the high abundance of the PorB porin in the OMV preparation, it did not appear to elicit high IgG levels in mice, perhaps arising from different immunogenic responses in mice and humans. Although we did record IgG reactivity against FetA in some human antisera, responses were generally weak, even though the parent strain for the OMV preparation was engineered to over-express the iron transporter^30^.

**Table 2:** Major IgG reactive antigens to human and mouse antisera following administration of MenPF-1 OMV vaccine^a^.

	NMBH4476^b^	Folded	iOMP?	Relative abundance in OMV^c^	IgG reactivity^d^	p-value^e^
**Human**						
PorA	0791	YES	YES	12,840	166 ± 27	5.93 × 10^−7^
RmpM	0378	YES	NO	1,970	149 ± 21	3.01 × 10^−8^
PorB	1982	YES	YES	13,870	114 ± 21	2.00 × 10^−6^
PilQ_25–777_	1758	YES	YES	850	68 ± 9	2.73 × 10^−9^
OpcA (D^f^)	1117	YES	YES	1,230	87 ± 26	1.42 × 10^−3^
OpcA (M^f^)	1117	YES	YES	1,230	68 ± 20	1.94 × 10^−3^
BamC	1243	YES	NO	340	53 ± 10	7.00 × 10^−6^
PilQ_25–580_	1758	YES	YES	850	29 ± 5	7.00 × 10^−3^
GNA1162 (M)	1046	YES	NO	180	14.8 ± 4.5	1.6 × 10^−2^
GNA1162 (D)	1046	YES	NO	180	14.1 ± 3.8	4.82 × 10^−3^
**Mouse**						
PorA	0791	YES	YES	12,840	509 ± 46	2.00 × 10^−6^
RmpM	0378	YES	NO	1,970	434 ± 17	2.75 × 10^−8^
LysM/M23	0741	YES	NO	ND^g^	187 ± 33	3.54 × 10^−4^
PilQ_25–580_	1758	YES	YES	850	106 ± 16	1.92 × 10^−4^
PilQ_25–777_	1758	YES	YES	850	114 ± 17	1.53 × 10^−4^

^a^Major reactive antigens are defined as p < 0.05.

^b^Proteins are represented as the last 4 digits of their NCBI protein accession number or gene numbers (NMBH4476_).

^c^Value represents the relative protein abundance in OMV as estimated by mass spectrometry.

^d^Difference in total IgG sero-reactivity between human pre-vaccination (T0) and post-3-vaccination (T20) sera or mouse naïve (N) and post-2-vaccination (T6) sera. The reactivity is given as arbitrary fluorescence measurement ± standard error.

^e^IgG reactivities at 0, 12 and 20 weeks were analysed for differences using one-way repeated measures ANOVA with Greenhouse-Greisser correction. For the mouse sera, p-values were determined by two-tailed T-test.

^f^M, monomer; D, dimer or multimer.

^g^Not detected.

As the strongest mean magnitude responses were observed at T20, we represented the enhanced IgG responses to individual antigens by calculating the increased relative response from T0 to T20. Seventy antigens showed an enhanced IgG reactivity (value ≥ 3) in more than one human vaccinee. Table 3 shows a summary for the seven major sero-reactive antigens identified in Table 2. Several of the highly responding antigens have been reported previously as capable of eliciting serum bactericidal activity and most are subject to some degree of phase or antigenic variation. More than 80% of recipients showed at least a 3-fold increase in IgG against PorA, PorB and the full length PilQ_25–777_ secretin. We identified NMBH4476_1243 as a homolog of BamC, which forms part of the outer membrane protein BAM assembly complex^42,43^: it showed an increase in IgG levels by a factor of at least 3 in 14 out of 25 recipients (56%). Other antigens induced a lower frequency of positive responses, specifically RmpM, the iOMP adhesin OpcA and a lipoprotein (GNA1162).

**Table 3:** Summary of individual vaccinee responses to antigens with high IgG reactivities.

Protein name	Gene name^a^	Function^b^	Individual vaccinee response to MenPF-1 OMV vaccinec																									SBA?^e^	Variability^f^	
			1	2	3	4	5	6	7	8	9	10	11	12	13	14	15	16	17	18	19	20	21	22	23	24	25		P	A
PorA	0791	Transport	<3	8	6	5	11	**5**	4	5	7	10	4	6	<3	27	10	5	6	7	<3	6	14	8	4	5	4	YES^76^	YES^77^	YES^78^
RmpM	0378	OM stability	4	7	5	<3	3	9	<3	8	13	16	5	4	3	19	15	5	<3	4	6	7	7	8	15	<3	3	YES^56^	NO^79^	NO^80^
PorB	1982	Transport	5	13	29	18	16	12	—	—	6	—	19	47	107	95	—	42	38	195	9	—	76	8	30	10	<3	YES^81^	NO^82^	YES^83^
PilQ_25–777_	1758	Type IV pilus formation	0	27	8	7	13	14	4	15	14	93	5	9	18	—	—	7	29	21	12	<3	5	3	14	13	3	YES^63^	NO^79^	NO^84^
OpcA (D)	1117	Adhesion	4	5	<3	5	<3	3	3	<3	3	<3	<3	4	<3	7	3	9	<3	5	<3	<3	<3	3	<3	<3	<3	YES^85^	YES^86^	NO^87^
OpcA (M)	1117	Adhesion	6	6	<3	3	<3	5	<3	<3	4	<3	4	3	<3	9	4	6	<3	3	<3	<3	4	3	<3	<3	3	YES^85^	YES^86^	NO^87^
BamC	1243	Protein assembly	—	—	18	<3	24	—	—	41	13	95	<3	<3	13	91	5	—	—	109	21	32	—	—	112	13	4	YES^43^	NO^43^	NO^43^
PilQ_25–580_	1758	Type IV pilus formation	3	5	4	3	<3	3	3	<3	11	10	<3	4	5	43	3	4	6	5	3	<3	4	3	4	<3	<3	YES^63^	NO^79^	NO^84^
GNA1162 (M)	1046	Unknown	3	5	<3	<3	<3	<3	<3	<3	<3	<3	<3	3	<3	5	<3	<3	<3	<3	3	<3	6	3	4	<3	<3	—	—	—
GNA1162 (D)	1046	Unknown	<3	<3	<3	<3	<3	3	<3	<3	<3	<3	<3	3	<3	5	<3	<3	<3	<3	<3	<3	5	4	<3	<3	<3	—	—	—

^a^Proteins are listed as the last 4 digits of their NCBI protein accession number or gene numbers (NMBH4476_).

^b^Protein function is detailed based on previous experimental findings or predictions from the Pfam database^75^.

^c^Values represent the proportional increase in serum reactivity to antigen i.e. (fluorescence value at T20)/(fluorescence value at T0). Where the T0 value was very low or zero, the cell is left blank.

^e^Published Serum Bactericidal Assay to a homolog.

^f^Phase (P) and antigenic (A) variability of antigens.

^g^M, monomer; D, dimer.

### Analysis of sequence conservation of selected antigens

PorA and PorB, are major meningococcal surface antigens, and are subject to a high degree of sequence variation, principally within the surface-exposed loop regions, probably as a result of immune selection pressure^44,45^. The degree of sequence variation within the other antigens in Table 1 and how it maps onto three-dimensional structure has been less well studied, however. OpcA is an iOMP with 10 membrane-spanning β-strands which is subject to much less sequence variation and has been advocated previously as a potentially valuable vaccine component^13,46,47^. We therefore set out to analyse the relationship between sequence and structure for the other antigens identified in Table 2.

**Figure 4:**
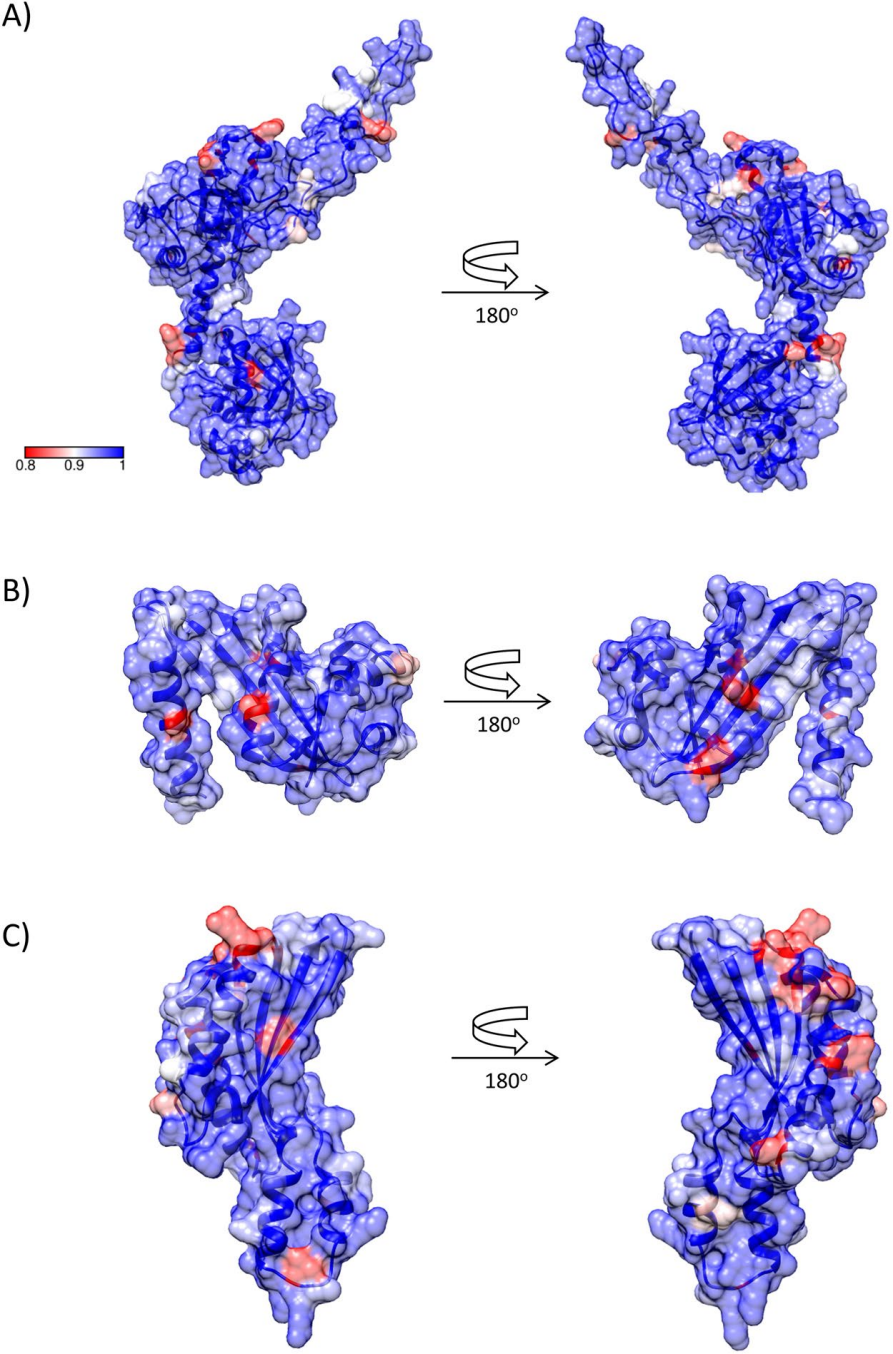
Structures and sequence variation of *N. meningitidis* BamC, GNA1162 and RmpM. (**A**) BamC. The model for BamC was generated using SWISS-MODEL^71^, based on PDB accession 5D0Q. (**B**) GNA1162 (PDB accession 4HRV). (**C**) RmpM (PDB accession 1R1M). Figures were generated using CONSURF^72^ and CHIMERA^73^. Surfaces are coloured by sequence conservation, from 80% or lower (red), through 90% (white) to 100% (blue).

BamC, GNA1162 and RmpM do not contain membrane-spanning β-strands and consist of α/β globular domain structures^41,42,48^. Using all available allele sequences from the Neisseria PubMLST databank^49^, sequence variation was mapped onto the crystal structures of GNA1162^48^ and RmpM^50^, and a molecular model of BamC, derived from the crystal structure of the BamACDE complex from *E. coli*^42^. Sequence variation was limited, with relatively few residues showing conservation of 80% or less (Fig. 4). There were no noticeable ‘hotspots’ of variation, with the possible exception of RmpM, which showed a marginally greater distribution at one end of the α/β domain (Fig. 4C).

**Figure 5:**
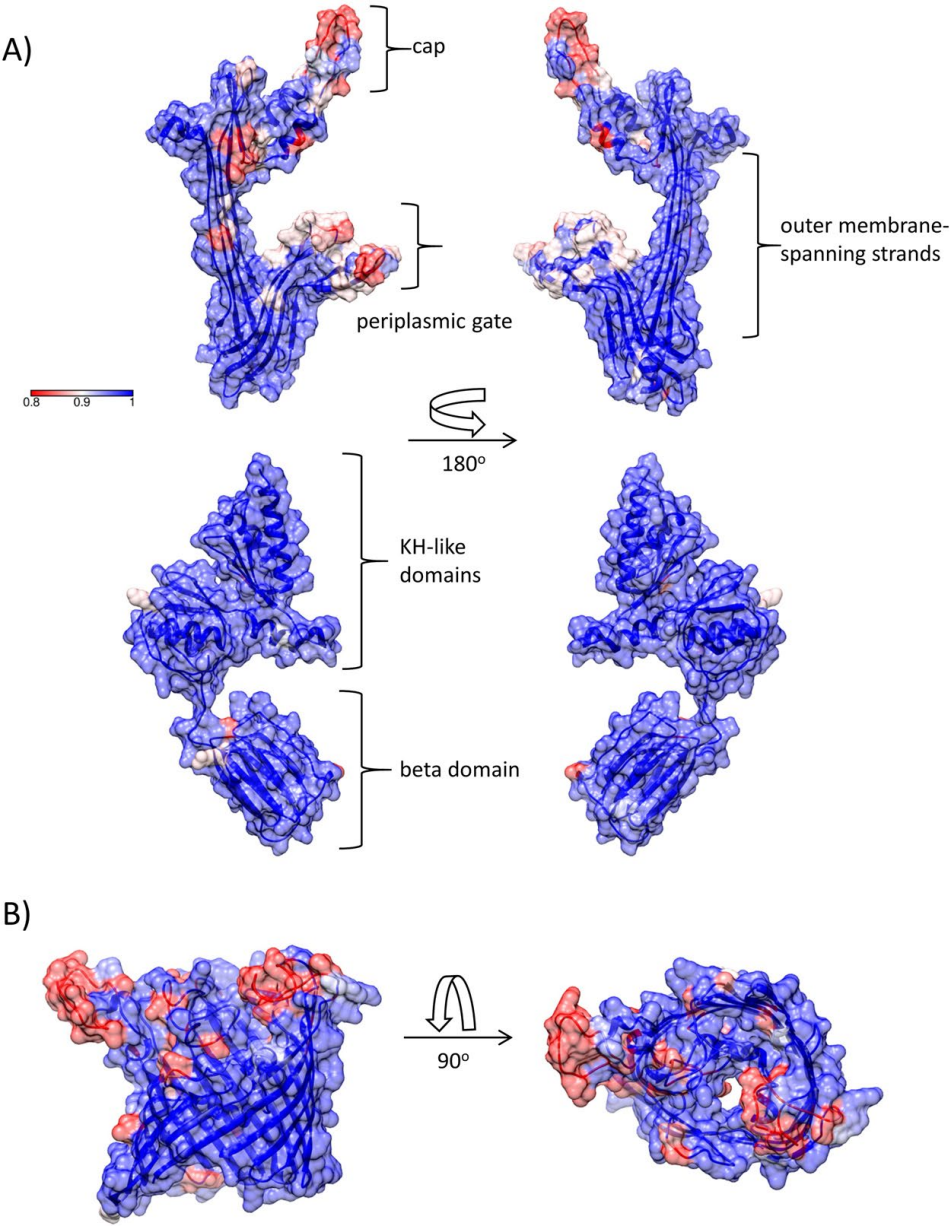
Structures and sequence variation of *N. meningitidis* PilQ and PorA. (**A**) PilQ. Upper panel: outer membrane-spanning domain; lower panel: periplasmic domains. The model for the membrane-spanning domain was generated by I-TASSER^74^ using multiple templates. The periplasmic domain was based on PDB accession 4AV2. (**B**) PorA. The model for PorA was generated using SWISS-MODEL^71^, based on PDB accession 3A2S.

In contrast, the PilQ secretin adopts a complex, multidomain oligomeric assembly which spans the outer membrane and part of the periplasm. Using recent structures derived from related secretins^51^, a model for the transmembrane portion of PilQ was generated; a previous NMR study generated structural models for the KH-like α/β domains and β domain which constitute the portion within the periplasm^52^. Sequence variation mapped to two parts of the transmembrane region, specifically the cap and periplasmic gate, which closes off the internal chamber within the oligomer^51^ (Fig. 5A). Sequence variation within the periplasmic domains was much more limited and not concentrated into any specific region. As a basis for comparison, we conducted a similar analysis on PorA, where antigenic variation has been studied in more detail. Sequence variation is concentrated into the surface-exposed loop regions, particularly loops 1 and 4 (Fig. 5B). This suggests that IgG recognition of PilQ is also concentrated into peptide sequences within the cap and periplasmic gate structures, an observation which could be of value in development of vaccines incorporating PilQ antigens.

## Discussion

OMV-based vaccines have been widely used since the 1970s, with reported efficacies above 70%^53^. A major limitation, however, is that protection against heterologous strains is limited^54^. This can be attributed, at least in part, to antigenic variation within the major outer membrane protein antigen, PorA^44^. However, OMVs represent a complex mixture of different antigens, several of which could assist in providing protection. For example, a study of antibody responses to an OMV vaccine based on strain H44/76 showed that the iOMP OpcA also made a contribution to bactericidal activity^55^. More generally, identification of the additional antigens which are at least able to elicit IgG responses will be valuable in identifying what further protective antigens there are in OMVs. The fact that we identify PorA as the most reactive antigen is to be expected, and provides an encouraging validation of our approach (Table 2). This was not the case with a similar antigen microarray study which used iOMPs in a denatured state^29^; this emphasises the importance of maintaining conformational epitopes in microarray screens.

Although our microarray contained over 90 proteins, only about 10% gave IgG responses in the human vaccinees which we could assign as significant. That is not to say that other proteins, or indeed non-protein components, do not play a role in providing protection in some vaccine individuals. It does suggest, however, that a panel of judiciously chosen antigens might be able to provide a level of protection similar to an OMV preparation, at least as far as the protein components of OMVs are concerned. Interestingly, we find that four out of the seven responding antigens in humans are iOMPs (Table 2). This observation poses a challenge for the development of antigen microarrays- iOMPs tend to be more difficult to isolate in their native conformational states than their soluble counterparts. Here, we used a generalized refolding protocol which was successful previously^11–13^, although it is imperfect: we have not been able to develop a refolding protocol which successfully reconstitutes the dodecameric PilQ assembly, for example. PilQ is a member of the secretin family, a group of iOMPs which are challenging to isolate and study^51^. The fact that our recombinant PilQ sample shows a well-defined peak in the Sypro Orange fluorescence assay suggests that some conformational epitopes are retained, however (Supplementary Fig. S2). This assay is a relatively crude measure of the folded state but provides a guide. Inevitably, protein antigen microarrays will be incomplete, to the extent that they omit proteins which are difficult to express, purify or reconstitute into their native conformations. Nevertheless, the fact that we identified several iOMPs (Table 2) suggests that we have been able to overcome these obstacles, at least to some extent.

The highest IgG responses observed in this study were directed against PorA, PorB and RmpM, which are thought to form a complex *in vivo*^41^. PorA and PorB form β-barrel structures, characteristic of bacterial porins, with surface-exposed loop regions^44^. RmpM, however, consists of a flexible N-terminal region and a C-terminal domain which binds to peptidoglycan^41,56^. The N-terminal region binds to PorA and PorB on the periplasmic side of the barrel^41,57^- this model suggests that no part of RmpM is surface-exposed in intact bacteria. This proposition is supported by earlier work which showed that antibodies derived from human OMV vaccinees reacted against segments of the protein which were not exposed in intact bacteria^56^. An obvious explanation is that portions of RmpM become exposed during OMV manufacture, which can stimulate an IgG response but, if correct, these epitopes are unlikely to be effective in providing protection.

An unanticipated outcome of this study was the identification of two lipoproteins which have received relatively little attention previously as potential meningococcal vaccine components. BamC is part of the BamABCDE (BAM) complex, which promotes insertion and folding of β-barrel iOMPs into the outer membrane^58^. Some components are dispensable for function in some organisms- BamE is not necessary for viability in *N. gonorrhoeae*, for example^59^. Delgado *et al*. identified NMBH4476_1243 (NCBI accession number) as a homolog of BamC from *E. coli*^43^. They showed that mice immunized with the BamC lipoprotein reconstituted into liposomes produced bactericidal antibodies which conferred protection in an infant rat model^43^. However, recent structural studies on the *E. coli* BAM complex suggest that BamC is likely located in the periplasm^42^. We also found sero-reactivity against BamA, although whether it appeared as meeting the significance criterion for inclusion in Table 2 depended on the statistical test employed. BamA is the key iOMP component of the BAM complex and the crystal structure of BamA from *N. gonorrhoeae* shows several external loop regions which could interact with antibody^60^. Further, a reverse vaccinology strategy applied to search for potential gonococcal vaccine candidates from naturally released gonococcal OMVs identified a BamA homolog which induced bactericidal antibodies^61^. BAM complex proteins are therefore worthy of further consideration as potential meningococcal vaccine components.

GNA1162 was originally identified as a potential meningococcal vaccine component using a reverse vaccinology approach^16^; FACS analysis suggested that it was surface-exposed, although serum bactericidal activity was weak (1/4). The crystal structure of GNA1162 has been determined, but there are few details available on its function: structural similarities were identified with TolB and LptE, suggestive of a role in transport or translocation^48^. Using the sequences from the Neisseria PubMLST databank^49^, we identified 426 alleles but, overall, a high degree of sequence conservation, with only a few residues exhibiting less than 80% sequence conservation (Fig. 4B). Given the strong IgG response to GNA1162 (Table 2), it may be worth revisiting the use of this lipoprotein as a useful vaccine component.

Antigens derived from the PilQ secretin also featured in the list of proteins reacting strongly against IgG from humans and mice (Table 2). PilQ forms a cage-like dodecameric assembly which spans the outer membrane and part of the periplasm^52,62^. It is a member of the secretin family of outer membrane proteins, and forms a large pore in the membrane for the passage of the assembled type IV pilus. Two PilQ constructs were used: one, PilQ_25–777_, constitutes the mature protein, whereas PilQ_25–580_ covers just the predicted periplasmic domains and omits the C-terminal, membrane-spanning region. Our findings are consistent with previous research which demonstrated that a 406–777 residue fragment of PilQ is both antigenic in mice and the antisera generated were bactericidal^63^.

Recent structural work on related secretins enabled us to construct models for the membrane-spanning and periplasmic PilQ domains. Modelling of the C-terminal region, which is the most conserved part of the multimer, enables us to identify which regions might potentially be surface-exposed (Fig. 5A). Mapping of the sequences from 992 alleles identified two ‘hotspots’ of variation within the cap and periplasmic gate regions; much less variation was seen within the periplasmic domains. One explanation for the higher sequence variation observed within these regions is that they are subject to immune selection pressure, in a similar fashion to PorA (Fig. 5B). Such a conclusion should be treated with caution, however: further evidence is required, such as the mapping of epitopes recognised by neutralising antibodies to these regions. Both the cap and periplasmic gate structures would be displaced on passage of an assembled pilus fibre (6–7 nm in diameter)^64^, and it is possible that this could accentuate external exposure of these parts of the secretin.

Two vaccines, 4C-MenB^65^ and rLP2086^66^, have been licensed for use against capsular group B meningococcal disease. 4C-MenB is a four component vaccine which contains two fusion recombinant proteins, NHBA-GNA1030 and fHbp-GNA2091, in combination with the adhesin NadA and a detergent-extracted OMV preparation derived from strain NZ98/254 (New Zealand). rLP2086 comprises two recombinant lipidated variants of fHbp (factor H binding protein), which has been a major focus of meningococcal vaccine antigen development^67^. Although these antigens were included in our protein microarray library, none showed an increased response after MenPF-1 vaccination, probably because they are present at low levels in the OMV preparation.

In summary, this work has shown that minor OMV antigens can elicit significant levels of IgG response and are therefore worthy of consideration as components in future vaccines.

## Methods

### MenPF-1 OMV vaccine

MenPF-1 vaccine is an OMV-based vaccine produced at Norwegian Institute of Public Health, (NIPH) from a genetically modified capsular *Neisseria meningitidis* capsular group B (NmB) strain H44/76 characterised as B:15: P1.7,16: F3-3:ST-32. The strain was genetically modified by replacing the wild-type *fetA* promoter, whose expression is dependent on iron concentration in the growth medium, with a *porA* promoter sequence to produce a modified meningococcal strain H44/76 which constitutively expresses FetA and retains wild-type expression of PorA^8,31^. The MenPF-1 vaccine consists of detergent-derived OMVs formulated with aluminium hydroxide, sucrose and water to contain 25 μg OMV proteins per 0.5 ml vial^30^.

### Serum samples from pre-clinical and clinical trials with MenPF-1 OMV vaccine

For this study, 10 C57BL/6 mice (female, 18–22 g) were immunised subcutaneously with 2 doses of 2.5 μg MenPF-1 vaccine. Each dose was 3 weeks apart and the terminal bleeds, collected 3 weeks after the second dose, were used for microarray analysis. This dosage and schedule had previously been shown to induce the production of PorA- and FetA-specific IgG responses^8^. All procedures were performed in accordance with the terms of the UK Home Office Animals Act Project License. Procedures were approved by the University of Oxford Animal Care and Ethical Review Committee.

Pre-clinical and Phase 1 clinical immunogenicity studies on MenPF-1 were carried out in mice and adult humans as described^8,30^. 52 volunteers (male and female, 18–50 yrs) were split equally into 2 dose groups to receive 25 μg of MenPF-1 vaccine in one group and 50 μg in the other. Three doses of vaccine were administered by intra-muscular injection 8 weeks apart and serum samples were collected 4 weeks after each dose^30^. Samples used for this study were pre-vaccination (T0), 12 (T12) and 20 (T20) weeks post-vaccination respectively. Ethical approval for the study was granted by NRES Oxford A (12/SC/0023) and the trial was registered with clinicaltrials.gov and EudraCT (NCT01640652 and 2012-001046-17 respectively). It was conducted in accordance with the principles of the Declaration of Helsinki (2008) and the International Conference on Harmonization (ICH) Good Clinical Practices standards.

### Protein characterization using multidimensional LC-MS/MS

The proteomics experiments were performed using a non-clinical GMP batch of MenPF vaccine (lot number FMOX1102) formulated with 3% sucrose to a final protein concentration of 0.05 mg/mL and Al(OH)_3_ concentration 3.3 mg/mL (~aluminium content of 1.15 mg/mL)^8^. The solution containing 600 µL of vaccine sample; 400 µL of 1 M tetraethylammonium bromide was added to a 1 mg RapiGest bottle (Waters, UK) and incubated at 100 °C for 5 min. When it reached ambient temperature (22 °C), the solution was transferred to a 20 µg trypsin bottle (Promega, UK) and placed at 37 °C overnight. The digestion was terminated and the RapiGest was disintegrated by addition of 5 µL trifluoroacetic acid and incubated at 22 °C for 30 min. The digest (~1 mL) was dried down under vaccum (using a Speedvac) to ~70 µL. Multidimensional LC-MS/MS was carried out according the method described by Chan *et al*.^68^. The 12 fractions collected by high pH RP-HPLC separation were each subjected to 240-min LC-MS/MS peptide sequencing using a MS system (Thermo Scientific) equipped with a nano-electrospray ion source and two mass detectors, linear trap (LTQ) and Orbitrap, coupled with an Ultimate 3000 nano-LC system. Data analysis including mass spectra processing and database searching was carried out using the Thermo Proteome Discoverer 1.4, with built-in Sequest (Thermo Scientific). Initial mass tolerances for protein identification by MS were set to 10 ppm. Up to two missed tryptic cleavages were considered and methionine oxidation was set as dynamic modification. Peptide sequences by MS/MS were only included when correlation scores were greater than 1.5, 2 or 2.2 for charge states 1, 2 and 3, respectively. An unambiguous identification was considered when at least two unique peptides matched to the protein. The protein FASTA databases were downloaded from www.uniprot.org, release 2014_07 including the complete entries from MenB H44/76 strain.

### Cloning, expression and purification of meningococcal OMPs

The open reading frames (ORFs) of selected OMPs of NmB strain were cloned into pOPINF and pOPINE plasmid vectors using a high-throughput In-Fusion™ cloning method developed by the Oxford Protein Production Facility (OPPF). The pOPINF plasmid encodes N-terminal hexa-histidine (His) tagged proteins while the pOPINE plasmid encodes C- terminal His tagged proteins^69^. The ORFs of gene encoding proteins were engineered to improve their solubility and expression. The gene sequences encoding mature NHBA, and NadA proteins, were synthesized *in vitro* (GeneArt, Invitrogen) and optimised for expression in *E. coli*. Synthesized genes were then cloned into pET22b(+) plasmid (Novagen) vector in between the *NdeI* and *XhoI* restriction sites to generate C-terminal hexa-histidine tagged proteins. All recombinant plasmids generated were sequenced (GATC Biotech) and confirmed with reference to the nucleotide database at the National Centre for Biotechnology Information^70^.

Recombinant plasmids were transformed into XL-10 Gold ultra-competent *E. co*li cells (Stratagene) for plasmid amplification and subsequently into BL21 (DE3) competent *E. coli* cells (New England Biolabs) for protein expression. Cultured cells were grown in terrific broth and induced at A_600_ (absorbance at 600 nm) of 0.8 by adding isopropyl-β-1-D-thiogalactopyranoside (IPTG; Sigma-Aldrich) to a final concentration of 0.5 mM for overnight expression at 16 °C.

1 mL aliquots of pre- and post-induction cell cultures were collected to assess the expression of target protein and its cellular location. Cells were pelleted by centrifugation at 16,000 × *g* for 5 min and subsequently, re-suspended in 500 μL of lysis buffer (50 mM HEPES-NaOH pH 7.5; 200 mM NaCl; 5% glycerol; 25 mM EDTA). The expression of target protein in total cell extract and soluble supernatant fractions from pre- and post-IPTG induction samples was analysed by SDS-PAGE. Proteins present in both total extract and supernatant fractions were regarded as soluble proteins and purified from the supernatant fraction. Proteins that were present only in the total extract samples were recovered from inclusion bodies (IBs). They were solubilised using urea and subsequently refolded using a protocol described previously^11^.

All solubilized target proteins were subject to sequential chromatographic steps for purification as described previously^11,34^. In brief, target proteins were purified with nickel iminodiacetic acid resin (Generon) using 50 mM HEPES pH7.5, 200 mM NaCl, 500 mM imidazole and 0.05% Glycerol for soluble proteins and 20 mM Tris-HCl pH 7.9; 0.5 M NaCl; 0.1% (v/v) LDAO 500 mM imidazole for refolded proteins. All eluents were analysed using 12% w/v SDS-PAGE (Invitrogen); fractions with target protein were pooled for further purification and concentrated using centrifugal concentrators (Sartorius) at 6,000 × *g*. To determine their oligomeric state, purified proteins were passed through a size exclusion chromatography column (Superdex S-75 or S-200 Hi-Load 16/60 column; GE Healthcare) on an AKTA-FPLC system (GE Healthcare). Column buffers used were 25 mM HEPES pH 7.5, 150 mM NaCl for soluble proteins and 20 mM Tris-HCl pH 7.9; 0.1 M NaCl; 0.1% (v/v) LDAO for refolded proteins. Eluted protein samples were analysed for purity by SDS-PAGE, and concentrated with Vivaspin® centrifugal concentrators (Sartorius). All purified OMPs were concentrated to 5–10 mg/ml, protein concentration estimated from absorbance at 280 nm and stored at −80 °C prior to microarray printing.

### Protein folding fluorescence temperature ramp assay

20 µL of each purified OMP was prepared at 25 µg/ml and 1x SYPRO® orange dye (Sigma) in appropriate buffer (25 mM HEPES-NaOH pH 7.5 and 150 mM NaCl for soluble proteins and 20 mM Tris-HCl pH 7.9, 0.5 M NaCl and 0.1% LDAO for refolded proteins). Samples were transferred to MicroAmp® Fast Optical 96 well reaction plates (Applied Biosystems) and sealed. The assay was conducted in a StepOne® Real Time PCR System (Life Technologies). Samples were heated from 25 to 95 °C at a rate of 1 °C/min. Fluorescence intensities were measured with excitation and emission wavelengths of 575/602 nm respectively. Samples were recorded in triplicate. Control wells consisting of buffer and dye only was used to normalise the fluorescence intensities for each protein. A reference ovalbumin protein sample was used as a positive control.

### Antigen microarray fabrication

The meningococcal protein microarray slides were custom-made by Arrayjet Limited, UK. Recombinant meningococcal protein samples were printed from 384 well plates containing 10 µL of each OMPs diluted to 1 µg/µL with Arrayjet printing buffer® (Arrayjet). Control spots consisted of a) monoclonal mouse and human IgG were prepared at a concentration of 0.3 µg/µL b) purified *E. coli* soluble lysate, *E. coli* IB extracts c) purified EBNA-1 viral protein at 0.1 µg/µL d) Arrayjet printing buffer® control spots. Two drops of 100 pL were arrayed per spot onto ONCYTE® SuperNOVA nitrocellulose coated glass slides (Grace Bio-Labs). The spot distance was set as 190 μm in the vertical and horizontal directions. Each microarray slide consisted of 16 identical blocks with each miniarray containing 91 OMP preparations and 5 control samples in triplicate. Printing was carried out at 18 °C and 50% relative humidity; arrayed protein slides were air-dried overnight at 4 °C and stored at −20 °C until used.

### Validation of protein spots on microarray slide

Protein spots were assessed for spot quality, circularity and consistency by using mouse monoclonal anti-tetra-histidine IgG antibody (Qiagen) to detect hexa-histidine tagged OMPs. Preliminary experiments with mouse and human sera showed that 1:125 dilutions of serum produced the best signal without significant background. Anti-mouse IgG Alexa Fluor 555 secondary antibody (Molecular Probes) was used to detect bound antibodies. Signal intensities were quantified and normalised against background using the GenePix® Pro 7 Microarray Image Analysis software (Molecular Devices).

### Microarray Immunogenicity probing

The Proplate® Multi-Array Slide System (Grace BioLabs) was used to create 16 leak-proof minipads on the meningococcal protein microarray slides; one minipad was used per serum sample. Minipads were blocked with 300 µL of 3% bovine serum albumin (Thermo Fisher) in Phosphate Buffered Saline (PBS) for 1 hr at room temperature. Addition of *E. coli* soluble lysate and inclusion body extracts to the serum sample were used to reduce non-specific recognition of *E. coli* proteins. 300 µl of 1:125 diluted serum in PBS supplemented with 10% w/v *E. coli* lysate (5 mg/mL) and IB extracts (5 mg/mL) were added per well and slides were incubated overnight at 4 °C under gentle agitation. After washing twice with 250 µL PBS-T (0.05% Tween-20) and once in PBS, for 10 min each time, slides were incubated in the dark with 300 µL of 1:1000 diluted anti-human or anti-mouse IgG Alexa Flour 555 secondary antibody (Molecular Probes), diluted in PBS, for 2 hr at 25 °C under gentle agitation. Wash steps were repeated as described above in the dark, then slides were rinsed in de-ionized water and dried by centrifugation at 200 × *g* for 2 min. Slides were scanned in a Gene Pix Pro 4200A microarray scanner (Molecular Devices) with the photo multiplier tube set to 100% for 532 nm wavelength and image analysis and data quantification was carried out using GenePix Pro 7 Microarray Image Analysis software (Molecular Devices).

### Data and statistical analysis

Microarray spot intensities were quantified using GenePix Pro 7 Microarray Image Analysis software (Molecular Devices) which utilises automatic background subtraction for each spot. The spot intensities for each protein were recorded in triplicate, arithmetic means determined and spot intensities for buffer-only-controls subtracted. To determine the increase in IgG reactivity for a particular antigen as shown in Table 2, the corrected value obtained from serum collected before immunization was subtracted from the same value obtained from serum after immunization (eg T20-T0 for human sera). A two-tailed T-test was used to calculate p-values and test for significance between doses per protein.

## Supplementary information

Supplementary Information
