# Supplementary material for: Immunogenicity profiling of protein antigens from capsular group B Neisseria meningitidis
Source: Sci Rep. 2019 May 2;9:6843. doi: 10.1038/s41598-019-43139-0 (PMC6497663; doi:10.1038/s41598-019-43139-0)
Supplement: Supplementary file 1 — Supplementary Information [file 41598_2019_43139_MOESM1_ESM.docx]

Immunogenicity profiling of protein antigens from capsular group B Neisseria meningitidis

Amaka M. Awanye, Chun-Mien Chang, Jun Wheeler, Hannah Chan, Leanne Marsay, Christina Dold, Christine S. Rollier, Louise Bird, Joanne E Nettleship, Raymond Owens , Andrew J. Pollard and Jeremy P. Derrick

**
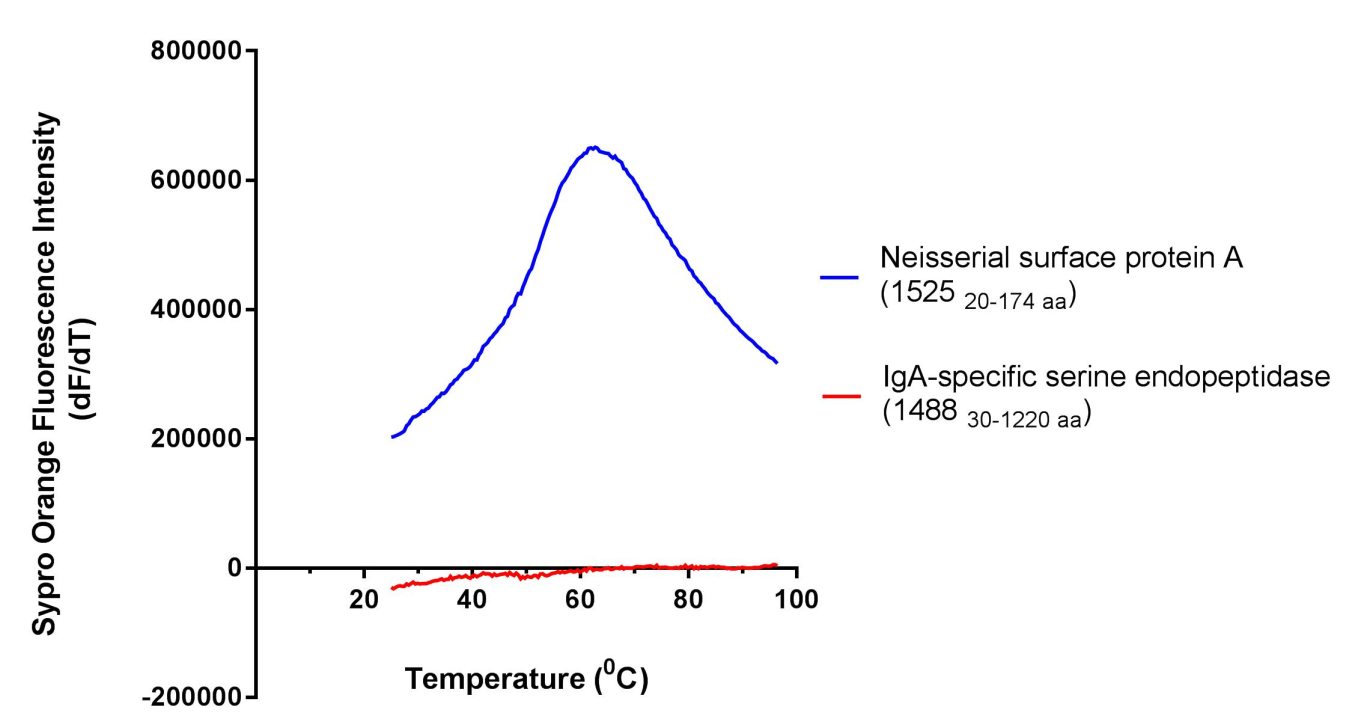
**

**Supplementary Fig. S1: Validation of protein folding by fluorescence temperature shift assay.** A Sypro Orange fluorescence temperature shift assay was used to evaluate the folded state of each purified protein. Two examples are shown: one, NspA monomer shows a well-defined transition temperature (Tm), whereas the IgA endopeptidase does not, and therefore is unlikely to retain tertiary structure.


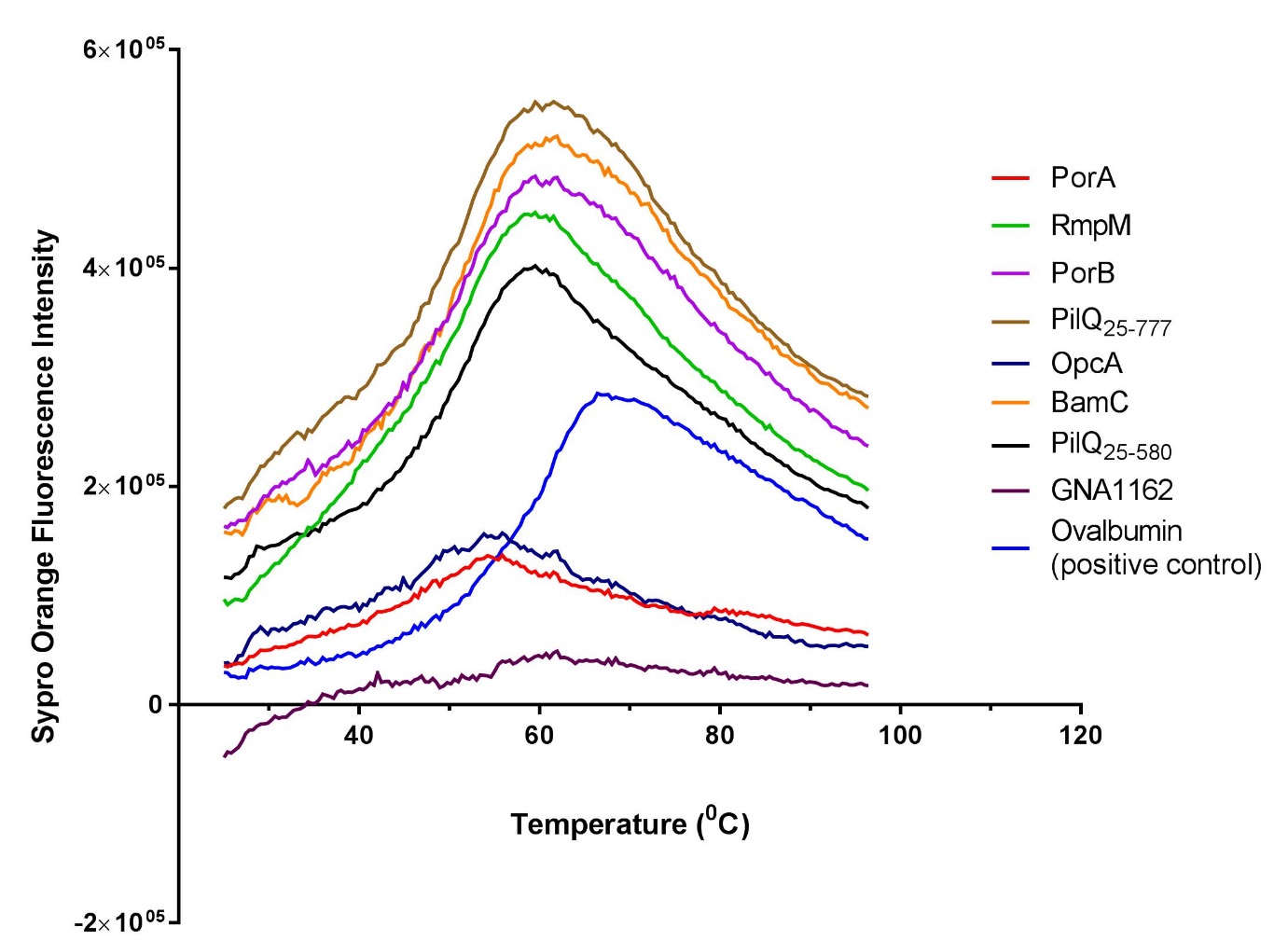


**Supplementary Fig. S2: Fluorescence temperature shift assay of proteins which show the strongest reactions against human IgG.** Fluorescence temperature shift assay was used to evaluate the folded state of each purified protein.

**Supplementary Table 1. Protein composition of a non-clinical GMP batch of MenPF vaccine.** Proteins were identified using multidimensional LC-MS/MS peptide sequencing and database searching. Major IgG reactive antigens, as identified in Table 2, are highlighted in yellow.

| **Accession** | **Description** | **Coverage**  **(%)** | **Number of unique Peptides sequenced** | **Number of peptide spectra matched** | **Score** |
| --- | --- | --- | --- | --- | --- |
| E6MVH3 | FetA | 68 | 51 | 5875 | 12849 |
| E6MZM0 | Major outer membrane protein P.IB (PorB) | 67 | 20 | 4856 | 13878 |
| E6MXW0 | Major outer membrane protein P.IA (PorA) | 69 | 21 | 3306 | 9187 |
| E6N0K3 | IgA-specific serine endopeptidase | 38 | 43 | 1364 | 3438 |
| E6MX18 | OmpA family protein (RmpM) | 38 | 15 | 1059 | 1979 |
| F0MJ06 | Iron(III) ABC transporter, periplasmic iron(III)-binding protein | 47 | 18 | 737 | 1639 |
| E6MXF3 | Outer membrane OpcA family protein | 41 | 12 | 584 | 1231 |
| E6MUV5 | 50S ribosomal protein L2 | 62 | 15 | 574 | 822 |
| F0MJW3 | Uncharacterized protein | 25 | 46 | 509 | 907 |
| E6MUW3 | 50S ribosomal protein L5 | 69 | 12 | 502 | 991 |
| E6MWK7 | CsgG family protein | 50 | 8 | 496 | 793 |
| Q9ZHF3 | Type IV pilus biogenesis and competence protein PilQ | 33 | 21 | 495 | 858 |
| E6N099 | Cysteine synthase | 55 | 15 | 483 | 1130 |
| F0MNM5 | 50S ribosomal protein L14 _NEIMH] | 61 | 9 | 445 | 995 |
| E6MXJ2 | Transferrin-binding protein 1 | 34 | 29 | 416 | 798 |
| E6MW18 | 50S ribosomal protein L19 | 51 | 5 | 406 | 894 |
| F0MMT2 | Iron receptor protein | 14 | 5 | 403 | 6 |
| E6MUP2 | 50S ribosomal protein L25 | 58 | 10 | 401 | 620 |
| E6MUY7 | Outer membrane protein assembly factor BamA | 38 | 26 | 396 | 681 |
| E6MUV3 | 50S ribosomal protein L4 | 52 | 9 | 392 | 864 |
| F0MM31 | Methyltransferase domain protein | 13 | 3 | 375 | 78 |
| E6MUU9 | 30S ribosomal protein S10] | 63 | 6 | 371 | 526 |
| F0MLH7 | Iron-regulated protein frpC | 22 | 4 | 355 | 751 |
| E6MZZ2 | tRNA-specific 2-thiouridylase MnmA | 11 | 4 | 355 | 14 |
| F0MJ54 | Iron-regulated protein FrpA | 20 | 2 | 354 | 755 |
| E6N0E1 | Ribosomal RNA large subunit methyltransferase E | 9 | 2 | 338 | 2 |
| E6MUW4 | 50S ribosomal protein L6 | 44 | 6 | 325 | 642 |
| F0MNM0 | 50S ribosomal protein L22 | 50 | 6 | 322 | 276 |
| F0MM85 | Hemagglutinin/hemolysin family protein | 22 | 35 | 322 | 154 |
| E6MVI6 | Outer membrane autotransporter barrel domain protein | 24 | 29 | 321 | 597 |
| F0MNN4 | 50S ribosomal protein L15 | 56 | 8 | 317 | 712 |
| F0MK72 | TonB-dependent hemoglobin receptor | 34 | 24 | 303 | 605 |
| E6MZP5 | 50S ribosomal protein L13 | 61 | 7 | 301 | 447 |
| E6MWE7 | Geranyltranstransferase | 17 | 2 | 299 | 2 |
| E6MYB6 | Filamentous hemagglutinin family N-terminal domain protein | 21 | 39 | 298 | 343 |
| E6MUV8 | 30S ribosomal protein S3 | 46 | 13 | 291 | 269 |
| F0MNN1 | 50S ribosomal protein L18 | 48 | 5 | 290 | 980 |
| F0MNN9 | 30S ribosomal protein S11 | 31 | 4 | 290 | 747 |
| E6MZ71 | DNA translocase FtsK | 10 | 3 | 284 | 194 |
| E6MZC1 | Aspartyl/glutamyl-tRNA(Asn/Gln) amidotransferase subunit B | 20 | 5 | 281 | 2 |
| E6MV96 | ATP synthase subunit beta | 35 | 12 | 273 | 593 |
| E6N0E3 | Delta-aminolevulinic acid dehydratase | 24 | 6 | 270 | 126 |
| E6MU96 | Elongation factor Tu | 31 | 10 | 264 | 496 |
| E6MVG3 | Adhesion and penetration protein | 22 | 26 | 262 | 463 |
| F0MNM2 | 50S ribosomal protein L16 | 49 | 5 | 262 | 325 |
| E6MU06 | DNA polymerase III, epsilon subunit | 20 | 4 | 260 | 2 |
| E6MZT2 | Phospholipid-binding domain protein | 51 | 8 | 253 | 672 |
| E6MZZ1 | AMP-binding enzyme family protein | 15 | 6 | 241 | 29 |
| E6MWB2 | Glutamate-ammonia-ligase adenylyltransferase | 10 | 4 | 240 | 2 |
| E6MXQ0 | Lactoferrin-binding protein A | 40 | 29 | 238 | 323 |
| E6MVN9 | Aspartate aminotransferase | 27 | 8 | 234 | 320 |
| F0MNL0 | 30S ribosomal protein S7 | 45 | 6 | 232 | 562 |
| F0MMW4 | Fimbrial protein | 31 | 1 | 222 | 710 |
| F0ML36 | Heme-utilization protein Hup | 31 | 20 | 221 | 300 |
| F0MIQ3 | Cell division protein ZipA | 36 | 9 | 218 | 295 |
| F0MIS3 | Conserved domain protein | 34 | 12 | 216 | 9 |
| F0MKT5 | Extracellular serine protease | 21 | 20 | 215 | 430 |
| F0MK28 | Multiple transferable resistance system protein MtrE | 45 | 16 | 215 | 429 |
| F0MMY6 | Putative lipoprotein (BamC) | 27 | 9 | 202 | 347 |
| E6MWN1 | Dihydroxy-acid dehydratase | 19 | 9 | 198 | 37 |
| E6MUE3 | DNA-binding protein | 48 | 3 | 198 | 2 |
| E6MV98 | ATP synthase subunit alpha | 30 | 12 | 197 | 286 |
| E6MWC1 | DNA helicase II | 12 | 4 | 191 | 5 |
| E6MZJ2 | Chaperone protein DnaJ | 12 | 6 | 188 | 29 |
| E6MWK5 | Putative lipoprotein (GNA1162) | 17 | 3 | 187 | 175 |
| E6MUA1 | DNA-directed RNA polymerase subunit beta GN=rpoB PE=3 SV=1 - [E6MUA1_NEIMH] | 27 | 28 | 187 | 34 |
| E6MXC8 | AsmA family protein | 15 | 8 | 187 | 21 |
| E6MY03 | Transketolase | 28 | 14 | 185 | 300 |
| E6N0K7 | Outer membrane protein assembly factor BamD | 54 | 12 | 181 | 331 |
| E6MV19 | 1-deoxy-D-xylulose-5-phosphate synthase | 13 | 2 | 179 | 10 |
| E6MU78 | Aspartate carbamoyltransferase | 16 | 1 | 178 | 41 |
| F0MLL6 | Ribosomal protein S2 | 65 | 12 | 176 | 446 |
| F0MIZ7 | MafB family protein | 34 | 13 | 174 | 28 |
| F0MID4 | Ribonuclease E | 34 | 22 | 170 | 195 |
| E6MUG4 | UPF0246 protein _1279 | 20 | 3 | 170 | 29 |
| E6N016 | Acetolactate synthase, small subunit | 55 | 7 | 169 | 79 |
| F0MNP0 | 30S ribosomal protein S4 | 46 | 9 | 168 | 288 |
| F0MNN8 | 30S ribosomal protein S13 | 50 | 7 | 164 | 375 |
| F0MIW8 | 50S ribosomal protein L28 | 51 | 4 | 164 | 100 |
| F0MK19 | Isoleucine--tRNA ligase | 11 | 6 | 164 | 2 |
| F0MNP2 | 50S ribosomal protein L17 | 71 | 7 | 162 | 245 |
| F0MLP6 | Uncharacterized protein | 16 | 17 | 162 | 170 |
| E6MV10 | Acetyl-CoA carboxylase, biotin carboxylase | 27 | 8 | 160 | 270 |
| E6MYW6 | ATP-dependent Clp protease ATP-binding subunit ClpA | 14 | 4 | 158 | 5 |
| E6MY76 | Cell division ATP-binding protein FtsE | 33 | 7 | 157 | 252 |
| E6MZZ6 | Glutathione synthetase | 18 | 6 | 157 | 238 |
| E6MVT4 | Aminomethyltransferase | 16 | 3 | 153 | 10 |
| F0MIL9 | DNA internalization-related competence protein ComEC/Rec2 | 17 | 9 | 151 | 2 |
| E6MXY4 | Protein RecA | 24 | 7 | 144 | 349 |
| E6MW84 | Glyceraldehyde-3-phosphate dehydrogenase, type I | 22 | 5 | 142 | 16 |
| E6MXI1 | Acetylglutamate kinase | 22 | 3 | 142 | 9 |
| E6MYC7 | Filamentous hemagglutinin family N-terminal domain protein | 19 | 6 | 141 | 159 |
| E6MY61 | Electron transfer flavoprotein subunit beta | 53 | 10 | 139 | 291 |
| E6MZ92 | C-terminal processing peptidase | 29 | 12 | 139 | 52 |
| E6N0H7 | CNP1 family protein | 30 | 3 | 139 | 5 |
| F0MNN2 | 30S ribosomal protein S5 | 48 | 5 | 134 | 226 |
| E6N0N1 | 50S ribosomal protein L20 | 28 | 4 | 134 | 157 |
| E6MZW4 | Argininosuccinate synthase | 17 | 5 | 133 | 61 |
| F0MIX0 | Ubiquinone biosynthesis hydroxylase, UbiH/UbiF/VisC/COQ6 family | 24 | 8 | 132 | 205 |
| E6MV21 | Fructose-bisphosphate aldolase, class II | 30 | 8 | 131 | 259 |
| E6MU97 | 50S ribosomal protein L11 | 52 | 5 | 131 | 75 |
| E6N017 | Acetolactate synthase | 24 | 10 | 131 | 16 |
| E6N037 | Alanine--tRNA ligase | 23 | 15 | 130 | 54 |
| F0MM11 | Ribonucleoside-diphosphate reductase | 22 | 13 | 128 | 72 |
| F0MJA8 | ABC transporter, ATP-binding protein | 29 | 13 | 127 | 242 |
| E6MY79 | Phosphoglycerate kinase | 28 | 10 | 127 | 161 |
| E6MUA3 | DNA-directed RNA polymerase subunit beta' | 21 | 23 | 127 | 82 |
| E6N045 | DNA topoisomerase 4 subunit A | 9 | 4 | 126 | 5 |
| E6MUL3 | N-acetylmuramoyl-L-alanine amidase | 28 | 10 | 125 | 176 |
| F0MNQ4 | Outer membrane protein, OmpH family | 40 | 6 | 123 | 164 |
| E6MWD5 | NADH dehydrogenase, G subunit | 13 | 5 | 123 | 14 |
| E6MWP1 | GTP-binding protein TypA/BipA | 16 | 10 | 122 | 216 |
| E6N0N4 | Cytosine-specific methyltransferase | 13 | 3 | 122 | 39 |
| E6MW09 | Hemolysin secretion/activation ShlB/FhaC/HecB family protein | 25 | 11 | 121 | 184 |
| E6MX38 | Delta-1-pyrroline-5-carboxylate dehydrogenase | 14 | 17 | 121 | 31 |
| E6MZP4 | 30S ribosomal protein S9 | 55 | 4 | 120 | 337 |
| F0MJK5 | Hemagglutinin/hemolysin family protein | 16 | 2 | 120 | 148 |
| E6MZT8 | Probable malate:quinone oxidoreductase | 14 | 4 | 118 | 73 |
| E6MZ93 | M23 peptidase domain protein | 39 | 23 | 116 | 96 |
| E6MWS5 | Homoserine dehydrogenase | 12 | 5 | 114 | 5 |
| E6MV02 | Carbamoyl-phosphate synthase large chain | 14 | 10 | 111 | 19 |
| E6MUX7 | Valine--tRNA ligase | 16 | 8 | 110 | 11 |
| E6MW49 | Phosphoenolpyruvate synthase | 19 | 10 | 109 | 90 |
| E6N0L4 | LPS-assembly lipoprotein LptE | 53 | 7 | 108 | 233 |
| E6MWZ6 | Phage Tail Collar domain protein | 12 | 3 | 107 | 50 |
| F0ML17 | Acetate kinase | 21 | 5 | 106 | 71 |
| E6MUW1 | 30S ribosomal protein S17 | 39 | 2 | 106 | 51 |
| E6MWV9 | Translation initiation factor IF-2 | 17 | 12 | 106 | 7 |
| F0MMW9 | Antioxidant, AhpC/TSA family/glutaredoxin | 45 | 9 | 104 | 163 |
| F0MLW6 | Acetyltransferase component of pyruvate dehydrogenase complex | 13 | 4 | 104 | 12 |
| E6N046 | His Kinase A domain protein | 11 | 4 | 103 | 39 |
| E6N094 | Polyribonucleotide nucleotidyltransferase | 26 | 13 | 102 | 160 |
| F0MJZ4 | Penicillin-binding protein 1A | 18 | 11 | 100 | 54 |
| F0MLB0 | Protein TldD | 29 | 6 | 100 | 5 |
| E6MZA1 | Pyruvate dehydrogenase E1 component | 12 | 10 | 99 | 123 |
| E6N0N7 | Phenylalanine--tRNA ligase beta subunit | 23 | 12 | 98 | 8 |
| F0MNB2 | Pantothenate synthetase | 28 | 5 | 97 | 11 |
| E6MZC7 | Exodeoxyribonuclease 7 large subunit | 18 | 4 | 97 | 5 |
| E6MVS2 | ApbE family protein | 9 | 3 | 97 | 2 |
| F0MNM9 | 30S ribosomal protein S8 | 45 | 5 | 95 | 68 |
| E6N0N5 | Type II restriction enzyme | 26 | 8 | 95 | 13 |
| E6MY66 | Glyceraldehyde-3-phosphate dehydrogenase, type I | 21 | 4 | 93 | 142 |
| F0MIT7 | LPS-assembly protein LptD | 23 | 12 | 93 | 105 |
| E6MWJ8 | Uncharacterized protein | 34 | 5 | 93 | 2 |
| E6MZT1 | Phosphoheptose isomerase | 15 | 2 | 92 | 2 |
| F0MNK2 | 50S ribosomal protein L1 | 52 | 7 | 91 | 164 |
| E6MU62 | Outer membrane protein, OMPP1/FadL/TodX family | 16 | 8 | 91 | 131 |
| E6N0F5 | Opacity protein opA50 | 33 | 6 | 91 | 68 |
| E6MYA9 | Anticodon nuclease | 23 | 5 | 90 | 2 |
| F0MNS2 | Dyp-type peroxidase family protein | 15 | 3 | 89 | 5 |
| F0MK15 | TonB-dependent siderophore receptor | 22 | 12 | 88 | 33 |
| E6MY31 | Glutamate dehydrogenase | 5 | 2 | 88 | 19 |
| E6MYV2 | Phosphoserine phosphatase SerB | 36 | 4 | 88 | 4 |
| E6MW94 | Oligopeptidase A | 17 | 12 | 87 | 2 |
| E6MY16 | Opacity protein | 39 | 10 | 86 | 114 |
| F0MIT8 | PPIC-type PPIASE domain protein | 44 | 13 | 85 | 132 |
| F0MLL8 | Uridylate kinase | 33 | 8 | 85 | 60 |
| F0MKF0 | Polyphosphate kinase | 16 | 8 | 85 | 19 |
| F0MMP7 | FAD binding/4Fe-4S binding/cysteine-rich domain protein | 16 | 14 | 85 | 7 |
| E6MYN4 | Tetratricopeptide repeat protein | 26 | 12 | 84 | 160 |
| F0MN40 | Ribose-phosphate pyrophosphokinase | 31 | 7 | 83 | 197 |
| F0MLW5 | Dihydrolipoyl dehydrogenase | 11 | 3 | 82 | 4 |
| E6MX68 | Cell division protein ftsA | 23 | 9 | 80 | 114 |
| E6MZE3 | ATP-dependent Clp protease ATP-binding subunit ClpX | 24 | 7 | 80 | 22 |
| F0MM95 | Ribonuclease R | 21 | 10 | 80 | 2 |
| E6MWX1 | UPF0210 protein _0575 | 13 | 4 | 79 | 21 |
| E6MUB8 | Hemagglutinin family protein | 21 | 8 | 79 | 7 |
| F0MIR7 | Putative lipoprotein, MafA family | 30 | 7 | 78 | 118 |
| E6MZ80 | 30S ribosomal protein S18 | 42 | 4 | 78 | 22 |
| F0ML54 | ATP-dependent RNA helicase HrpA | 14 | 9 | 78 | 5 |
| E6N012 | Aconitate hydratase 2 | 19 | 11 | 77 | 59 |
| E6MU42 | Protein yhgF | 19 | 11 | 77 | 9 |
| E6MYP9 | 50S ribosomal protein L21 | 36 | 2 | 76 | 180 |
| E6N033 | Uncharacterized protein | 30 | 4 | 76 | 69 |
| E6MWA6 | 3-oxoacyl-[acyl-carrier-protein] synthase 2 | 19 | 4 | 74 | 104 |
| F0MJG7 | Oxidoreductase, zinc-binding dehydrogenase family | 38 | 7 | 72 | 188 |
| E6MWL7 | Uncharacterized protein | 21 | 2 | 72 | 2 |
| E6MWT9 | ABC transporter family protein | 24 | 8 | 71 | 49 |
| F0MNB0 | LysM domain protein | 18 | 4 | 70 | 94 |
| E6MU64 | Pyruvate kinase | 21 | 8 | 70 | 85 |
| F0MLA5 | Phosphoenolpyruvate carboxylase | 17 | 12 | 70 | 7 |
| E6MXU7 | Lipoprotein | 22 | 2 | 70 | 2 |
| E6MUQ6 | Type IV pilus assembly protein PilX | 38 | 4 | 69 | 143 |
| E6MVY8 | Fun | 17 | 8 | 68 | 87 |
| F0MM22 | Acyl-CoA dehydrogenase domain protein | 16 | 7 | 68 | 68 |
| F0MNK0 | Transcription termination/antitermination protein NusG | 24 | 4 | 67 | 58 |
| E6MVJ3 | Arginine biosynthesis bifunctional protein ArgJ | 16 | 6 | 67 | 19 |
| E6MU34 | PilT | 21 | 6 | 66 | 120 |
| E6MU91 | DNA topoisomerase 1 | 16 | 5 | 66 | 2 |
| E6MYP8 | 50S ribosomal protein L27 | 36 | 4 | 65 | 119 |
| E6MUW6 | 50S ribosomal protein L30 | 49 | 3 | 65 | 86 |
| E6MYV0 | NAD(P) transhydrogenase subunit alpha | 17 | 6 | 65 | 67 |
| F0MKP9 | Glycine--tRNA ligase beta subunit | 16 | 8 | 65 | 7 |
| E6MY68 | DNA mismatch repair protein MutS | 15 | 4 | 65 | 4 |
| E6MZA5 | Uncharacterized protein | 20 | 6 | 65 | 2 |
| E6MVC0 | Stringent starvation protein A | 22 | 2 | 64 | 154 |
| E6MV36 | TonB-dependent siderophore receptor | 22 | 11 | 64 | 2 |
| E6MVQ9 | Chaperone protein DnaK | 26 | 9 | 63 | 46 |
| E6MUA8 | Elongation factor G | 19 | 9 | 63 | 25 |
| E6N0M0 | RNA polymerase sigma factor RpoH | 20 | 4 | 63 | 7 |
| F0MKX2 | ATP-dependent DNA helicase | 19 | 3 | 63 | 3 |
| E6N020 | Uncharacterized protein | 19 | 4 | 62 | 93 |
| F0MM03 | 30S ribosomal protein S1 | 22 | 9 | 62 | 36 |
| E6N088 | Tyrosine recombinase XerD | 29 | 3 | 62 | 2 |
| F0MNH9 | Transposase, IS30 family | 30 | 1 | 62 | 2 |
| E6N0C8 | Exodeoxyribonuclease V, beta subunit | 12 | 8 | 62 | 2 |
| E6MW24 | Putative transcriptional regulatory protein CpxR | 10 | 3 | 60 | 125 |
| E6MW59 | Peptide chain release factor 3 | 9 | 3 | 60 | 34 |
| E6MUT2 | Leukotoxin secretion protein D | 24 | 8 | 60 | 2 |
| E6MZG7 | Helix-turn-helix domain, rpiR family protein | 24 | 4 | 59 | 131 |
| E6MU85 | Ribosomal RNA small subunit methyltransferase B | 11 | 3 | 59 | 39 |
| E6MUX0 | DNA-directed RNA polymerase subunit alpha | 19 | 6 | 58 | 66 |
| F0MJQ5 | Phospholipase A1 | 24 | 6 | 58 | 15 |
| E6MXT5 | 3-isopropylmalate dehydratase large subunit | 9 | 5 | 58 | 7 |
| E6MVT3 | Bkd operon transcriptional regulator | 19 | 2 | 58 | 2 |
| E6MZ32 | Enolase | 25 | 8 | 57 | 125 |
| E6MWD9 | NADH-quinone oxidoreductase subunit J | 16 | 3 | 57 | 17 |
| F0MKA3 | Type IV pilus assembly protein PilC | 20 | 6 | 57 | 9 |
| E6MU31 | Neisseria PilC family protein | 18 | 2 | 57 | 7 |
| E6MZZ8 | Glycerol-3-phosphate regulon repressor | 16 | 2 | 57 | 5 |
| E6MWB8 | Biotin-dependent carboxylase domain protein | 15 | 3 | 57 | 2 |
| F0MJR6 | Uncharacterized protein | 10 | 3 | 57 | 2 |
| F0MNL7 | 50S ribosomal protein L23 | 31 | 3 | 56 | 154 |
| E6N044 | 2,3-bisphosphoglycerate-dependent phosphoglycerate mutase | 22 | 4 | 56 | 52 |
| E6MXH2 | DNA translocase FtsK | 10 | 6 | 56 | 26 |
| E6N0R9 | Fimbrial protein P9-2 | 37 | 4 | 56 | 22 |
| E6N0L3 | Uncharacterized protein | 8 | 2 | 56 | 5 |
| F0MK29 | Transposase, IS30 family | 26 | 1 | 56 | 2 |
| E6MWY8 | Uncharacterized protein | 22 | 5 | 56 | 2 |
| E6MZR6 | Biotin-(Acetyl-CoA-carboxylase) ligase/transcriptional activator, Baf family | 16 | 4 | 56 | 2 |
| E6MV27 | Orotate phosphoribosyltransferase | 26 | 3 | 55 | 139 |
| F0MLY2 | 30S ribosomal protein S6 | 43 | 4 | 55 | 70 |
| E6MVD0 | Uncharacterized protein | 22 | 2 | 55 | 4 |
| E6MUX3 | Site-determining protein | 28 | 3 | 54 | 33 |
| E6MXW9 | Phospholipase D family protein | 26 | 8 | 54 | 5 |
| E6N021 | Histidinol dehydrogenase | 18 | 8 | 53 | 95 |
| E6N006 | Peptidyl-prolyl cis-trans isomerase | 34 | 9 | 53 | 88 |
| E6N0S6 | Glutamine--fructose-6-phosphate aminotransferase [isomerizing] | 18 | 8 | 53 | 66 |
| E6MVB5 | Transglycosylase SLT domain protein | 25 | 12 | 53 | 34 |
| F0MLT4 | L-lactate dehydrogenase | 12 | 2 | 53 | 10 |
| E6MX69 | Cell division protein FtsZ | 21 | 6 | 52 | 93 |
| E6MV75 | 3-oxoacyl-[acyl-carrier-protein] synthase 3 | 18 | 5 | 52 | 78 |
| F0MM47 | Phosphoribosylformylglycinamidine cyclo-ligase | 23 | 4 | 52 | 43 |
| E6MUV2 | 50S ribosomal protein L3 | 37 | 5 | 51 | 109 |
| E6MZZ0 | CTP synthase | 16 | 8 | 51 | 46 |
| F0MMI0 | 2-isopropylmalate synthase | 21 | 8 | 51 | 28 |
| E6MV91 | Glycosyl transferase family 2 family protein | 19 | 5 | 51 | 26 |
| E6MUQ9 | Inner membrane protein | 13 | 9 | 51 | 4 |
| E6MWN3 | Sulfite reductase [NADPH] flavoprotein alpha-component | 3 | 3 | 51 | 4 |
| E6MYD8 | ATP-dependent DNA helicase RecG | 11 | 5 | 51 | 2 |
| E6MU33 | Twitching motility family protein | 14 | 4 | 50 | 17 |
| E6MXS7 | 3-isopropylmalate dehydrogenase | 19 | 4 | 50 | 14 |
| E6MW65 | Phosphate acetyltransferase | 18 | 7 | 49 | 76 |
| E6MYR2 | Branched-chain amino acid aminotransferase | 18 | 4 | 49 | 37 |
| E6MUB0 | Replication initiation factor | 17 | 4 | 49 | 2 |
| E6MWJ3 | Thiol:disulfide interchange protein | 17 | 2 | 48 | 53 |
| E6N070 | Glutathione peroxidase | 40 | 5 | 48 | 16 |
| E6MVZ0 | Phosphatidylserine decarboxylase proenzyme | 28 | 6 | 48 | 14 |
| E6MY60 | Electron transfer flavodomain protein | 16 | 4 | 47 | 78 |
| F0MNG8 | Uncharacterized protein | 19 | 1 | 47 | 50 |
| E6MUQ1 | Superoxide dismutase | 14 | 2 | 47 | 32 |
| E6MXB8 | Serine--tRNA ligase | 23 | 7 | 47 | 7 |
| F0ML62 | Pseudouridine synthase | 24 | 3 | 47 | 2 |
| E6N0Q7 | UDP-3-O-[3-hydroxymyristoyl] N-acetylglucosamine deacetylase | 13 | 3 | 46 | 10 |
| E6MWA0 | Catalase | 14 | 7 | 46 | 5 |
| E6MWH6 | Putative exonuclease | 10 | 3 | 46 | 2 |
| E6MZT5 | Uncharacterized protein | 32 | 3 | 45 | 93 |
| E6N0R6 | Fimbrial protein | 43 | 2 | 45 | 22 |
| E6MVZ6 | Zonula occludens toxin family protein | 12 | 4 | 45 | 6 |
| E6N0F9 | DNA ligase | 15 | 5 | 45 | 4 |
| E6MTY8 | SsrA-binding protein | 30 | 4 | 44 | 55 |
| E6MUM9 | Aminopeptidase N | 15 | 9 | 44 | 19 |
| E6MVV4 | tRNA-specific adenosine deaminase | 9 | 2 | 44 | 7 |
| E6MVB3 | ABC transporter family protein | 28 | 4 | 43 | 4 |
| E6MZ46 | LysM domain protein | 16 | 7 | 42 | 87 |
| E6MW22 | Acetyltransferase, GNAT family | 9 | 6 | 42 | 31 |
| E6MXV7 | Metallopeptidase family M24 family protein | 5 | 4 | 42 | 12 |
| E6MZD3 | Methylase | 31 | 7 | 42 | 3 |
| E6N0Q2 | DNA repair protein RecN | 15 | 5 | 42 | 2 |
| F0MLD5 | Single-stranded DNA-binding protein | 49 | 6 | 41 | 67 |
| E6MXL1 | Uncharacterized protein | 28 | 5 | 41 | 27 |
| E6MUM0 | Putative ATP-dependent RNA helicase rhlE | 21 | 8 | 41 | 25 |
| E6MZ35 | Ribonucleoside-diphosphate reductase 1 subunit beta | 5 | 1 | 41 | 16 |
| E6MVK9 | Uncharacterized protein | 21 | 6 | 41 | 15 |
| F0MJK7 | Initiator repB protein family | 30 | 3 | 41 | 2 |
| E6N035 | Putrescine-binding periplasmic protein | 17 | 6 | 40 | 10 |
| E6MZN7 | RmuC domain protein | 24 | 5 | 40 | 2 |
| E6MZX1 | Outer membrane protein, OMP85 family | 18 | 8 | 39 | 59 |
| E6MZD5 | Putative ATP-dependent RNA helicase rhlE | 24 | 7 | 39 | 34 |
| E6MX91 | Guanosine-3',5'-bis(Diphosphate) 3'-pyrophosphohydrolase | 14 | 9 | 39 | 9 |
| F0MKU8 | DNA polymerase I | 14 | 8 | 39 | 5 |
| E6MVE1 | Aminodeoxychorismate synthase | 7 | 4 | 39 | 4 |
| F0MNI4 | Peptide deformylase | 28 | 4 | 38 | 37 |
| E6MZ54 | Cytidylate kinase | 24 | 3 | 38 | 6 |
| E6MU27 | Replication initiation factor family protein | 31 | 4 | 38 | 2 |
| E6MUV6 | 30S ribosomal protein S19 | 18 | 2 | 37 | 47 |
| E6MVE7 | 60 kDa chaperonin | 30 | 11 | 37 | 28 |
| E6MWP6 | Inosine-5'-monophosphate dehydrogenase | 15 | 4 | 37 | 17 |
| E6N013 | Ornithine carbamoyltransferase | 15 | 3 | 37 | 2 |
| E6MW81 | Outer membrane protein assembly factor BamE | 38 | 5 | 36 | 58 |
| E6MXJ7 | 30S ribosomal protein S20 | 47 | 4 | 36 | 55 |
| F0MN50 | Methionine--tRNA ligase | 17 | 7 | 36 | 11 |
| E6MVZ8 | IS110 family transposase | 13 | 5 | 36 | 2 |
| E6MZQ9 | Thiamine-phosphate synthase | 32 | 4 | 35 | 61 |
| E6MV01 | Uncharacterized protein | 23 | 4 | 35 | 30 |
| E6MXK4 | Biosynthetic arginine decarboxylase | 9 | 6 | 35 | 15 |
| E6MW90 | DNA gyrase subunit B | 7 | 4 | 35 | 3 |
| E6MZ02 | Type III restriction-modification system EcoPI enzyme res | 10 | 6 | 35 | 3 |
| F0MJG3 | Putative thiol:disulfide interchange protein DsbC | 25 | 5 | 34 | 53 |
| E6MVB0 | Lipoprotein | 37 | 8 | 34 | 47 |
| F0MLZ2 | Trigger factor | 29 | 7 | 34 | 42 |
| E6N0D8 | Peptidyl-tRNA hydrolase | 21 | 2 | 34 | 42 |
| E6N0T9 | ABC transporter periplasmic binding , thiB subfamily protein | 18 | 4 | 34 | 37 |
| E6MWI3 | Uncharacterized protein | 17 | 3 | 34 | 28 |
| E6MYY4 | GTPase Der | 10 | 4 | 34 | 22 |
| F0MM23 | Lytic murein transglycosylase B | 20 | 4 | 34 | 14 |
| E6MWN7 | Siroheme synthase | 17 | 5 | 34 | 13 |
| E6MU94 | Methyltransferase family protein | 12 | 2 | 34 | 2 |
| F0MJK3 | Uncharacterized protein | 16 | 3 | 34 | 2 |
| E6N0H6 | Acetyl-coenzyme A carboxylase carboxyl transferase subunit beta | 20 | 5 | 33 | 23 |
| E6MWM0 | Acetyl-coenzyme A carboxylase carboxyl transferase subunit alpha | 18 | 3 | 33 | 17 |
| E6MZG5 | Glucose-6-phosphate isomerase | 19 | 7 | 33 | 3 |
| E6MVY5 | Succinyl-CoA ligase [ADP-forming] subunit beta | 19 | 5 | 32 | 40 |
| E6N0J9 | Ribosomal RNA small subunit methyltransferase A | 18 | 3 | 32 | 14 |
| E6MUQ5 | Prepilin-type N-terminal cleavage/methylation domain protein | 14 | 3 | 32 | 7 |
| F0MMT4 | UvrABC system protein A | 18 | 8 | 32 | 2 |
| E6MZJ0 | dTDP-4-dehydrorhamnose 3,5-epimerase | 36 | 5 | 32 | 2 |
| E6MV82 | 3-oxoacyl-(Acyl-carrier-protein) reductase | 29 | 4 | 31 | 36 |
| F0MNH0 | Adenylosuccinate synthetase | 21 | 7 | 31 | 32 |
| E6MYE9 | S-adenosylmethionine synthase | 17 | 6 | 31 | 28 |
| E6N0S0 | Fimbrial MS11-D1 domain protein | 31 | 1 | 31 | 22 |
| E6MZH5 | Alcohol dehydrogenase, zinc-containing | 12 | 2 | 31 | 20 |
| E6MXE0 | Glycosyl transferases group 1 family protein | 21 | 6 | 31 | 16 |
| E6MW33 | (R,R)-butanediol dehydrogenase | 18 | 5 | 31 | 12 |
| F0MMA0 | Phosphoadenosine phosphosulfate reductase | 13 | 2 | 31 | 9 |
| E6N0L6 | Membrane protein | 37 | 6 | 31 | 8 |
| F0MM69 | Lon protease | 17 | 7 | 31 | 5 |
| F0MMU4 | Succinate dehydrogenase iron-sulfur subunit | 26 | 5 | 31 | 4 |
| E6MXY7 | ATP-dependent DNA helicase Rep | 16 | 10 | 31 | 2 |
| E6N031 | 4-carboxymuconolactone decarboxylase family protein | 28 | 1 | 31 | 2 |
| F0MJG4 | Macrolide export ATP-binding/permease protein MacB | 10 | 4 | 31 | 2 |
| E6MXB3 | tRNA/tmRNA (uracil-C(5))-methyltransferase | 28 | 7 | 30 | 34 |
| E6N0R4 | Fimbrial protein | 19 | 1 | 30 | 22 |
| F0MKH3 | Transcription termination/antitermination protein nusA | 24 | 9 | 30 | 11 |
| E6MY74 | Antioxidant, AhpC/TSA family | 53 | 5 | 30 | 8 |
| E6MXE6 | Glutamate dehydrogenase | 19 | 6 | 30 | 3 |
| E6MZ87 | Sel1 repeat family protein | 13 | 5 | 30 | 2 |
| E6MWN5 | Sulfate adenylyltransferase, small subunit | 15 | 3 | 30 | 2 |
| E6MVY6 | Succinyl-CoA ligase [ADP-forming] subunit alpha | 12 | 3 | 29 | 59 |
| E6MYT3 | Lipopolysaccharide ABC transporter, periplasmic lipopolysaccharide-binding protein | 23 | 3 | 29 | 40 |
| E6MXL4 | Uncharacterized protein | 22 | 6 | 29 | 32 |
| E6MZ42 | 1-acyl-sn-glycerol-3-phosphate acyltransferase | 14 | 3 | 29 | 24 |
| E6MZ37 | Cytosine-specific methyltransferase | 27 | 7 | 29 | 24 |
| E6MYY3 | Recombination-associated protein RdgC | 20 | 5 | 29 | 15 |
| E6MUN8 | Tetratricopeptide repeat family protein | 16 | 7 | 29 | 11 |
| E6N0I5 | Ribonuclease 3 | 24 | 3 | 29 | 9 |
| F0MMB3 | tRNA(Ile)-lysidine synthase | 12 | 3 | 29 | 7 |
| E6MU16 | Putative membrane protein | 8 | 2 | 29 | 2 |
| E6MVS8 | Na(+)-translocating NADH-quinone reductase subunit A | 14 | 3 | 29 | 2 |
| E6N058 | Fumarate hydratase | 14 | 5 | 28 | 14 |
| E6MWS7 | DNA-binding protein HU-beta | 28 | 2 | 27 | 78 |
| E6MWR2 | SPFH domain / Band 7 family protein | 13 | 4 | 27 | 32 |
| F0ML14 | Phytoene/squalene synthetase family protein | 23 | 4 | 27 | 31 |
| F0MID1 | tRNA uridine 5-carboxymethylaminomethyl modification enzyme MnmG | 21 | 11 | 27 | 4 |
| F0MNR0 | CRS1 / YhbY domain protein | 44 | 2 | 27 | 2 |
| F0MJG5 | Macrolide-specific efflux protein MacA | 13 | 3 | 27 | 2 |
| E6MYJ0 | 4-hydroxy-3-methylbut-2-enyl diphosphate reductase | 22 | 2 | 27 | 2 |
| E6MWT7 | Peptidyl-prolyl cis-trans isomerase | 19 | 6 | 27 | 2 |
| E6MXU2 | Copper-translocating P-type ATPase | 8 | 4 | 27 | 2 |
| E6MVC7 | AhpC/TSA family protein | 19 | 3 | 26 | 44 |
| E6MYF9 | Type IV pilus assembly PilM family protein | 8 | 3 | 26 | 43 |
| F0ML91 | Hypoxanthine phosphoribosyltransferase | 34 | 6 | 26 | 27 |
| F0ML03 | Azurin | 24 | 4 | 26 | 18 |
| E6MU43 | Capsule polysaccharide export ATP-binding protein CtrD | 18 | 2 | 26 | 14 |
| E6N0U3 | Signal recognition particle receptor FtsY | 20 | 4 | 26 | 12 |
| E6MXX6 | O-methyltransferase family protein | 15 | 2 | 26 | 10 |
| E6MUM2 | tRNA-dihydrouridine synthase | 9 | 3 | 26 | 8 |
| F0MLH3 | Lipid A biosynthesis (KDO)2-(Lauroyl)-lipid IVA acyltransferase | 17 | 4 | 26 | 8 |
| F0ML73 | Ubiquinone biosynthesis O-methyltransferase | 29 | 6 | 26 | 7 |
| F0MNU2 | Elongation factor 4 | 12 | 5 | 26 | 3 |
| F0MIE5 | L-serine ammonia-lyase | 12 | 3 | 26 | 3 |
| F0MIR6 | MafB family protein | 27 | 3 | 26 | 2 |
| F0MM26 | Long-chain-fatty-acid--CoA ligase | 15 | 6 | 25 | 17 |
| F0MM86 | Putative lipoprotein | 10 | 2 | 25 | 7 |
| E6MVS4 | Na(+)-translocating NADH-quinone reductase subunit F | 11 | 2 | 25 | 4 |
| E6MZB4 | NOL1/NOP2/sun family protein | 11 | 4 | 25 | 4 |
| F0MLP3 | Transferrin binding protein-like solute binding family protein | 15 | 5 | 25 | 4 |
| E6MVU2 | Electron transfer flavoprotein-ubiquinone oxidoreductase | 12 | 4 | 25 | 3 |
| E6MZ84 | Thioredoxin reductase | 10 | 3 | 25 | 2 |
| F0MKQ4 | ATP synthase F1, gamma subunit | 20 | 5 | 24 | 30 |
| E6MX45 | Thiol:disulfide interchange protein | 17 | 3 | 24 | 27 |
| E6MVY1 | Oxoglutarate dehydrogenase (Succinyl-transferring), E1 component | 8 | 6 | 24 | 21 |
| E6MYR0 | 2,3,4,5-tetrahydropyridine-2,6-dicarboxylate N-succinyltransferase | 26 | 7 | 24 | 18 |
| F0MLW8 | Proline--tRNA ligase | 21 | 9 | 24 | 17 |
| E6MWG9 | Phosphotransferase enzyme family protein | 11 | 4 | 24 | 13 |
| E6MY27 | ATP-dependent chaperone ClpB | 13 | 8 | 24 | 11 |
| E6MY47 | Carbon starvation protein A | 8 | 3 | 24 | 7 |
| E6MUT1 | Outer membrane efflux family protein | 16 | 7 | 24 | 2 |
| F0MMR5 | Bifunctional purine biosynthesis protein PurH | 14 | 5 | 23 | 12 |
| F0MJY5 | Serine-type D-Ala-D-Ala carboxypeptidase | 19 | 4 | 23 | 4 |
| E6MVP5 | Chromosome partition protein Smc | 12 | 14 | 23 | 2 |
| E6MZG8 | Glucokinase | 15 | 5 | 22 | 32 |
| E6N0E6 | Nitroreductase family protein | 21 | 4 | 22 | 21 |
| E6MXX3 | Iron-sulfur cluster binding protein | 12 | 5 | 22 | 16 |
| F0MK48 | DAHP synthetase family, class I | 26 | 5 | 22 | 5 |
| E6N0M4 | Conserved domain protein | 29 | 2 | 22 | 5 |
| F0MNU0 | Twitching motility protein PilT | 21 | 6 | 22 | 4 |
| E6MYN7 | M23 peptidase domain protein | 13 | 2 | 22 | 2 |
| E6MXL5 | Small GTP-binding domain protein | 30 | 4 | 21 | 32 |
| E6MVB6 | 30S ribosomal protein S21 | 30 | 1 | 21 | 26 |
| F0MK55 | D-lactate dehydrogenase, fermentative | 14 | 3 | 21 | 13 |
| E6MX15 | Transcriptional activator protein anr | 20 | 5 | 21 | 6 |
| E6MWH9 | Adenylosuccinate lyase | 11 | 2 | 21 | 2 |
| F0MLP9 | Phospholipase, patatin family | 22 | 5 | 20 | 37 |
| E6MYI6 | Esterase family protein | 10 | 2 | 20 | 14 |
| E6MYQ7 | Bacterial type II secretion system protein F domain protein | 17 | 4 | 20 | 8 |
| E6MZ06 | CobW family protein | 19 | 4 | 20 | 6 |
| E6MY84 | 6-phosphogluconate dehydrogenase, decarboxylating | 12 | 4 | 20 | 4 |
| E6MYG2 | Pilus assembly , PilP family protein | 12 | 2 | 20 | 2 |
| E6MUD2 | tRNA-dihydrouridine synthase | 22 | 2 | 19 | 2 |
| E6MZP2 | Putative GTP cyclohydrolase 1 type 2 | 10 | 2 | 19 | 2 |
| F0MLL3 | Uncharacterized protein | 34 | 5 | 19 | 2 |
| F0MNK3 | 50S ribosomal protein L10 | 30 | 3 | 18 | 41 |
| E6MZR9 | Bifunctional protein FolD | 25 | 5 | 18 | 25 |
| E6MVM9 | Endonuclease III | 14 | 3 | 18 | 5 |
| F0MJ71 | Na(+)-translocating NADH-quinone reductase subunit C | 19 | 2 | 18 | 5 |
| E6MXC6 | Dihydropteroate synthase | 19 | 3 | 18 | 4 |
| F0MMZ2 | CMP-N-acetylneuraminate-beta-galactosamide-alpha-2,3-sialyltransferase | 14 | 5 | 18 | 3 |
| E6MW48 | Transcription termination factor Rho | 15 | 4 | 18 | 2 |
| E6MY99 | Adenylate kinase | 19 | 2 | 18 | 2 |
| E6MX02 | Uncharacterized protein | 30 | 4 | 18 | 2 |
| F0MNP3 | Probable septum site-determining protein MinC | 14 | 3 | 17 | 19 |
| E6MV64 | Membrane protein insertase YidC | 11 | 3 | 17 | 15 |
| E6MUL2 | RNA methylase family protein | 13 | 5 | 17 | 11 |
| E6N034 | Putative membrane protein | 10 | 1 | 17 | 5 |
| E6MWT2 | Exodeoxyribonuclease V, alpha subunit | 19 | 7 | 17 | 2 |
| E6MYS2 | Uncharacterized protein | 22 | 3 | 17 | 2 |
| F0MNT6 | Uncharacterized protein | 44 | 4 | 17 | 2 |
| E6MUB7 | Uncharacterized protein | 38 | 2 | 17 | 2 |
| F0MK22 | Acetyltransferase PglI | 11 | 6 | 17 | 2 |
| E6MZW5 | Uncharacterized protein | 23 | 2 | 16 | 20 |
| E6MU24 | Ribonuclease PH | 29 | 5 | 16 | 17 |
| E6N0T2 | Imelysin family protein | 15 | 4 | 16 | 15 |
| E6MX50 | Ribosomal RNA small subunit methyltransferase H | 14 | 4 | 16 | 13 |
| E6MYG1 | Pilus assembly , PilO family protein | 23 | 3 | 16 | 11 |
| F0MKN1 | ATP phosphoribosyltransferase | 14 | 2 | 16 | 4 |
| E6MVX0 | 5-methyltetrahydropteroyltriglutamate--homocysteine methyltransferase | 15 | 5 | 16 | 3 |
| F0MNK9 | 30S ribosomal protein S12 | 45 | 7 | 16 | 3 |
| E6MWF0 | Putative ribosome biogenesis GTPase RsgA | 22 | 6 | 16 | 2 |
| E6MZF4 | Cysteine desulfurase IscS | 12 | 3 | 15 | 24 |
| E6N023 | Imidazoleglycerol-phosphate dehydratase | 24 | 6 | 15 | 13 |
| F0MMY3 | 23S rRNA (guanosine-2'-O-)-methyltransferase RlmB | 25 | 5 | 15 | 10 |
| E6N0Q5 | Uncharacterized protein | 14 | 1 | 15 | 7 |
| E6MY86 | NAD kinase | 10 | 2 | 14 | 31 |
| E6MY32 | Phosphoglycolate phosphatase | 14 | 2 | 14 | 25 |
| E6MU21 | ThiF family protein | 15 | 3 | 14 | 23 |
| E6MW37 | Protein-export membrane protein SecF | 10 | 2 | 14 | 17 |
| E6MW15 | ABC transporter, periplasmic substrate-binding protein | 12 | 3 | 14 | 16 |
| F0MKC4 | Factor H binding protein | 12 | 2 | 14 | 15 |
| E6MZB1 | RNA methyltransferase, TrmH family, group 1 | 13 | 3 | 14 | 13 |
| F0MJ28 | Putrescine ABC transporter, ATP-binding protein PotG | 12 | 4 | 14 | 9 |
| E6MXK5 | Agmatinase | 12 | 2 | 14 | 9 |
| E6N0D3 | Phosphoglucomutase | 21 | 5 | 14 | 7 |
| E6MVN5 | Magnesium and cobalt efflux protein CorC | 12 | 3 | 14 | 5 |
| E6MUQ2 | Replicative DNA helicase | 12 | 4 | 14 | 4 |
| E6MWX0 | Alanine racemase | 19 | 3 | 14 | 2 |
| E6N0B1 | Uracil phosphoribosyltransferase | 14 | 2 | 13 | 27 |
| E6MVW9 | Methylenetetrahydrofolate reductase | 15 | 4 | 13 | 14 |
| F0MKF2 | Chromosomal replication initiator protein DnaA | 7 | 4 | 13 | 12 |
| E6MW21 | 30S ribosomal protein S16 | 23 | 2 | 13 | 10 |
| E6MZ61 | AFG1-like ATPase family protein | 8 | 2 | 13 | 7 |
| E6MX23 | Aldose 1-epimerase | 16 | 5 | 13 | 5 |
| E6MU49 | Alpha-2,8-polysialyltransferase SiaD | 4 | 2 | 13 | 4 |
| E6N072 | Copper-containing nitrite reductase | 9 | 3 | 13 | 4 |
| E6N0T4 | Dyp-type peroxidase family protein | 12 | 3 | 13 | 4 |
| E6MZK0 | Ribosomal RNA large subunit methyltransferase H | 29 | 2 | 13 | 3 |
| E6MYQ5 | Dephospho-CoA kinase | 25 | 4 | 13 | 2 |
| E6MWT3 | Lipoprotein releasing system, transmembrane protein, LolC/E family | 11 | 5 | 13 | 2 |
| E6MVD1 | Toluene tolerance family protein | 10 | 2 | 12 | 20 |
| E6MVM8 | Protease Do | 9 | 2 | 12 | 17 |
| E6MWQ1 | Bacterioferritin | 29 | 4 | 12 | 17 |
| E6MWC8 | NADH-quinone oxidoreductase subunit B | 24 | 3 | 12 | 12 |
| F0MLH2 | Crossover junction endodeoxyribonuclease RuvC | 22 | 3 | 12 | 10 |
| E6MVW0 | Elongation factor P | 23 | 4 | 12 | 9 |
| F0MN53 | MltA family protein | 13 | 4 | 12 | 8 |
| F0MIK4 | Ferrochelatase | 10 | 3 | 12 | 7 |
| E6MZT0 | UPF0102 protein _2035 | 36 | 3 | 12 | 7 |
| F0MN84 | Capsule polysaccharide export outer membrane protein CtrA | 26 | 6 | 12 | 5 |
| E6MZS6 | GTPase Obg | 15 | 2 | 12 | 5 |
| E6MWX7 | Uncharacterized protein | 21 | 2 | 12 | 4 |
| F0MIC8 | Ribosomal RNA small subunit methyltransferase G | 21 | 2 | 12 | 3 |
| E6MWS3 | ABC transporter family protein | 16 | 6 | 12 | 2 |
| E6MUY3 | Acyl-[acyl-carrier-protein]--UDP-N-acetylglucosamine O-acyltransferase | 8 | 2 | 12 | 2 |
| E6MVC9 | Lipoprotein, VacJ family | 14 | 3 | 11 | 20 |
| E6MZP1 | Ubiquinol-cytochrome c reductase iron-sulfur subunit | 21 | 3 | 11 | 17 |
| E6N0P8 | UPF0042 nucleotide-binding protein _1448 | 14 | 3 | 11 | 16 |
| E6MZW2 | Peptidase family S49 family protein | 16 | 2 | 11 | 10 |
| F0MK51 | Membrane protein, dedA family | 17 | 2 | 11 | 10 |
| E6MZU4 | Elongation factor Ts | 25 | 4 | 11 | 9 |
| E6MX16 | HTH-type transcriptional regulator cysB | 6 | 2 | 11 | 5 |
| E6MYY1 | Deoxycytidine triphosphate deaminase | 23 | 4 | 11 | 4 |
| E6MUL6 | Glutamate racemase | 29 | 5 | 11 | 4 |
| E6MU79 | Aspartate carbamoyltransferase regulatory chain | 16 | 2 | 11 | 2 |
| E6MZS3 | Exodeoxyribonuclease III | 38 | 5 | 11 | 2 |
| E6MUT5 | Uncharacterized protein | 33 | 3 | 11 | 2 |
| E6MWK8 | Oxidoreductase, short chain dehydrogenase/reductase family | 19 | 2 | 10 | 16 |
| F0MKA4 | Carbamoyl-phosphate synthase small chain | 10 | 3 | 10 | 15 |
| E6MY46 | Lipoprotein | 17 | 2 | 10 | 12 |
| E6MZE5 | Ribosome-binding factor A | 33 | 3 | 10 | 7 |
| F0MK77 | HAD hydrolase, IIB family | 13 | 2 | 10 | 5 |
| E6MUL0 | Hydrolase, NUDIX family protein | 22 | 3 | 10 | 2 |
| F0MMJ2 | Gamma-glutamyltransferase | 6 | 4 | 10 | 2 |
| E6MU37 | Pyrroline-5-carboxylate reductase | 23 | 4 | 10 | 2 |
| E6MZ11 | Uncharacterized protein | 8 | 3 | 10 | 2 |
| F0ML07 | Uncharacterized protein | 12 | 3 | 10 | 2 |
| E6MVL4 | Periplasmic protein | 20 | 3 | 10 | 2 |
| E6N0Q6 | Ubiquinone/menaquinone biosynthesis C-methyltransferase UbiE | 9 | 2 | 9 | 17 |
| E6MUG3 | Aminotransferase, classes I and II | 10 | 2 | 9 | 10 |
| F0MNV1 | Phosphoribosylaminoimidazole-succinocarboxamide synthase | 16 | 3 | 9 | 2 |
| F0MJH5 | Endoribonuclease YbeY | 15 | 2 | 8 | 14 |
| E6MUR0 | Membrane fusion protein MtrC | 11 | 4 | 8 | 9 |
| E6MZT7 | Adhesin component | 35 | 3 | 8 | 6 |
| F0MK37 | 3-oxoacyl-(Acyl carrier protein) synthase II | 17 | 1 | 8 | 2 |
| F0MLR9 | Glucose-6-phosphate 1-dehydrogenase | 16 | 3 | 8 | 2 |
| E6N0P3 | ATP-dependent dethiobiotin synthetase BioD | 11 | 2 | 8 | 2 |
| E6MUX4 | Cell division topological specificity factor | 28 | 2 | 7 | 10 |
| F0MMM5 | Uncharacterized protein | 21 | 2 | 7 | 9 |
| E6MWB6 | Uncharacterized protein | 17 | 2 | 7 | 8 |
| E6MU88 | DNA-binding response regulator, Fis family | 6 | 2 | 7 | 5 |
| E6N0S4 | Glycerate dehydrogenase | 11 | 2 | 7 | 5 |
| E6MZF6 | FeS cluster assembly scaffold IscU | 27 | 2 | 7 | 4 |
| E6MXM8 | Phage uncharacterized protein | 8 | 2 | 7 | 2 |
| E6MYX1 | Membrane protein | 12 | 2 | 6 | 9 |
| E6MZD9 | Uncharacterized protein | 18 | 2 | 6 | 6 |
| F0MN24 | Prepilin-type N-terminal cleavage/methylation domain protein | 16 | 3 | 6 | 6 |
| E6MUK9 | Holo-[acyl-carrier-protein] synthase | 17 | 2 | 6 | 5 |
| E6MWZ9 | Conserved domain protein | 21 | 4 | 6 | 5 |
| E6MYZ9 | Anthranilate phosphoribosyltransferase | 7 | 2 | 6 | 2 |
| E6MYF5 | Cytochrome c family protein | 14 | 3 | 6 | 2 |
| E6N065 | Phosphomethylpyrimidine kinase | 16 | 3 | 6 | 2 |
| F0MLP8 | Peptide chain release factor 2 | 18 | 3 | 6 | 2 |
| E6MVJ2 | LrgB family protein | 9 | 2 | 5 | 10 |
| E6MUU1 | Chromosome partitioning protein, ParA family | 8 | 2 | 5 | 6 |
| E6N0C4 | Uncharacterized protein | 11 | 2 | 5 | 5 |
| E6MVG0 | Uncharacterized protein | 9 | 2 | 5 | 3 |
| E6MY22 | Putative lipoprotein | 38 | 2 | 5 | 3 |
| E6MZR5 | Sporulation related domain protein | 13 | 3 | 5 | 2 |
| E6MZP0 | Cytochrome b | 7 | 2 | 5 | 2 |
| E6MWG8 | DSBA-like thioredoxin domain protein | 16 | 2 | 4 | 8 |
| F0MML3 | 3-isopropylmalate dehydratase small subunit | 10 | 2 | 4 | 7 |
| E6MXL7 | Roadblock/LC7 domain protein | 29 | 3 | 4 | 7 |
| E6MZD2 | Thioredoxin | 15 | 2 | 4 | 5 |
| F0MIZ0 | YCII domain protein | 27 | 2 | 4 | 5 |
| E6MYQ4 | Type IV-A pilus assembly ATPase PilB | 6 | 3 | 4 | 2 |
| E6N069 | Muramoyltetrapeptide carboxypeptidase | 8 | 3 | 4 | 2 |
| E6MZB9 | Glutamyl-tRNA(Gln) amidotransferase subunit A | 10 | 3 | 4 | 2 |
| E6MWQ0 | Bacterioferritin | 15 | 2 | 2 | 2 |

**Supplementary Table S2:** Library of purified meningococcal proteins indicating protein fragment expressed, predicted molecular mass in kDa, oligomeric and conformational states.

|  |  |  |  |  |  |  |  |  |
| --- | --- | --- | --- | --- | --- | --- | --- | --- |
| **S/N** | **^¥^ GENE NAME (NMBH4476_)** | **PROTEIN NAME** | **MOL WT (kDa)** | **FRAGMENT LENGTH** | **^§^ BUFFER** | **OLIGOMERIC STATE** | **MELT CURVE RESULT** | **REFOLDED ?** |
| **1** | **0018** | Fimbrial protein | 14.7 | 30 - 167 aa | A + 5% glycerol | MONOMER | FOLDED | NO |
| **2** | **0043** | Peptide methionine sulfoxide reductase | 57.9 | 2 - 522 aa | A | OCTAMER | FOLDED | NO |
| **3** | **0043** | Peptide methionine sulfoxide reductase | 57.9 | 2 - 522 aa | A | TRIMER | FOLDED | NO |
| **4** | **0086** | Lipoprotein | 34.7 | 20 - 338 aa | A | MONOMER | FOLDED | NO |
| **5** | **0088** | Outer membrane protein, OMPP1/FadL/TodX family | 48.0 | 26 - 466 aa | D | DIMER | FOLDED | YES |
| **6** | **0103** | LysM domain protein | 44.0 | 23 - 405 aa | A | MONOMER | FOLDED | NO |
| **7** | **0178** | Outer membrane protein, OmpH family | 19.1 | 24 - 166 aa | A | MONOMER | FOLDED | NO |
| **8** | **0178** | Outer membrane protein, OmpH family | 19.0 | 2 - 166 aa | A | MONOMER | INCONCLUSIVE | YES |
| **9** | **0179** | Outer membrane protein BamA family | 88.3 | 22 - 797 aa | D | MONOMER | FOLDED | YES |
| **10** | **0199** | Outer membrane protein assembly factor BamE | 12.0 | 20 - 125 aa | A | MONOMER | FOLDED | NO |
| **11** | **0273** | DSBC thiosulfide | 28.6 | 31 - 260 aa | A | Both DIMER | FOLDED | NO |
| **12** | **0276** | PPIC-type PPIASE domain Protein | 38.5 | 22 - 360 aa | A | MONOMER | FOLDED | NO |
| **13** | **0289** | DSBA Thioredoxin domain Protein | 25.4 | 24 - 231 aa | A | MONOMER | FOLDED | NO |
| **14** | **0331** | Enoyl-[acyl-carrier-protein] reductase [NADH] | 27.7 | 2 - 261 aa | A | TETRAMER | FOLDED | NO |
| **15** | **0340** | Hypothetical protein: peptidyl proline isomerase ? | 31.5 | 21 - 288 aa | A | MONOMER | FOLDED | NO |
| **16** | **0341** | Conserved Hypothetical Protein | 28.7 | 2 - 252 aa | A | DIMER | FOLDED | NO |
| **17** | **0372** | Putative Lipoprotein MafA family | 34.0 | 27 - 313 aa | C | DECAMER | FOLDED | NO |
| **18** | **0372** | Putative Lipoprotein MafA family | 34.0 | 27 - 313 aa | C | MONOMER | FOLDED | NO |
| **19** | **0378** | RmpM | 26.2 | 23 - 242 aa | D | MONOMER | FOLDED | YES |
| **20** | **0399** | DSBA thioredoxin domain protein | 24.0 | 22 - 214 aa | A | MONOMER | FOLDED | NO |
| **21** | **0453** | Transferrin Binding Protein 2 | 74.1 | 26 - 691 aa | A + 5% glycerol | MONOMER | INCONCLUSIVE | NO |
| **22** | **0453** | Transferrin Binding Protein 2 | 74.1 | 26 - 691 aa | A + 5% glycerol | DIMER | INCONCLUSIVE | NO |
| **23** | **0454** | Transferrin Binding Protein 1 | 102.2 | 25 - 915 aa | D | DIMER | FOLDED | YES |
| **24** | **0455** | Putative putrescene ABC transporter, periplasmic putrescine-binding protein | 41.3 | 19 - 380 aa | A | DIMER | FOLDED | NO |
| **25** | **0455** | Putative putrescene ABC transporter, periplasmic putrescine-binding protein | 41.3 | 19 - 380 aa | A | MONOMER | FOLDED | NO |
| **26** | **0455** | Putative putrescene ABC transporter, periplasmic putrescine-binding protein | 41.3 | 24 - 380 aa | A | MONOMER | FOLDED | NO |
| **27** | **0507** | (MtrE) Multiple transferable Resistance System Protein | 50.5 | 2 - 467 aa | D | TRIMER | FOLDED | YES |
| **28** | **0507** | (MtrE) Multiple transferable Resistance System Protein | 50.5 | 2 - 467 aa | D | DIMER | FOLDED | YES |
| **29** | **0554** | Ton B Dependent Hemoglobin Receptor | 89.3 | 25 - 792 aa | D | DIMER | FOLDED | YES |
| **30** | **0591** | Opacity protein | 26.0 | 2 - 232 aa | D | MONOMER | FOLDED | YES |
| **31** | **0615** | Amino acid ABC transporter, periplasmic amino acid-binding protein | 29.0 | 26 - 268 aa | A | MONOMER | FOLDED | NO |
| **32** | **0630** | PotF1 (Putative Putrescine Periplasmic Binding Protein) | 41.9 | 2 - 376 aa | D | TETRAMER | FOLDED | YES |
| **33** | **0630** | PotF1 (Putative Putrescine Periplasmic Binding Protein) | 41.9 | 2 - 376 aa | D | DIMER | FOLDED | YES |
| **34** | **0652** | Ahpc/TSA antioxidant | 23.2 | 20 - 217 aa | A | MONOMER | FOLDED | NO |
| **35** | **0664** | Macrophage Infectivity Potentiator (MIP) | 28.9 | 23 - 272 aa | A | TRIMER | FOLDED | NO |
| **36** | **0674** | Putative uncharacterized protein | 15.2 | 20 - 157 aa | A + 5% glycerol | MONOMER | FOLDED | NO |
| **37** | **0695** | Azurin | 16.3 | 24 - 183 aa | A + 5% glycerol | MONOMER | INCONCLUSIVE | NO |
| **38** | **0730** | Heme-utilization protein Hup | 103.3 | 2 - 915 aa | D | MONOMER | FOLDED | YES |
| **39** | **0741** | LysM domain/M23 peptidase domain protein | 40.3 | 27 - 415 aa | A | MONOMER | FOLDED | NO |
| **40** | **0791** | PorA | 40.1 | 20 - 392 aa | D | MONOMER | FOLDED | YES |
| **41** | **0820** | Superoxide dismutase [Cu-Zn] | 19.5 | 23 - 186 aa | A | DIMER | INCONCLUSIVE | NO |
| **42** | **0986** | Homoserine dehydrogenase | 46.5 | 2 - 435 aa | A | MONOMER | INCONCLUSIVE | NO |
| **43** | **1044** | CsgG domain membrane protein | 21.9 | 22 - 223 aa | D | MONOMER | FOLDED | YES |
| **44** | **1044** | CsgG domain membrane protein | 23.7 | 2 - 223 aa | D | MONOMER | FOLDED | YES |
| **45** | **1046** | Putative Lipoprotein | 22.9 | 22 - 215 aa | D | TRIMER | FOLDED | YES |
| **46** | **1046** | Putative Lipoprotein | 22.9 | 22 - 215 aa | D | MONOMER | FOLDED | YES |
| **47** | **1114** | Gamma-glutamyltransferase | 64.9 | 2 - 606 aa | D | MONOMER | FOLDED | NO |
| **48** | **1117** | Outer membrane OpcA family protein | 28.1 | 20 - 272 aa | D | MONOMER | FOLDED | YES |
| **49** | **1117** | Outer membrane OpcA family protein | 28.1 | 20 - 272 aa | D | DIMER | FOLDED | YES |
| **50** | **1129** | SCP-like extracellular family protein | 48.0 | 21 - 426 aa | A | MONOMER | FOLDED | NO |
| **51** | **1139** | YceI family protein (GNA1030) | 20.4 | 20 - 187 aa | A | DIMER | FOLDED | NO |
| **52** | **1139** | YceI family protein (GNA1030) | 20.4 | 20 - 187 aa | A | MONOMER | FOLDED | NO |
| **53** | **1216** | Dihydrolipoyl Dehydrogenase | 50.1 | 2 - 477 aa | A | DIMER | FOLDED | NO |
| **54** | **1243** | Putative lipoprotein BamC homologue | 43.7 | 2 - 398 aa | D | MONOMER | FOLDED | YES |
| **55** | **1304** | Outer membrane protein LolB | 21.1 | 17 - 193 aa | D | OCTAMER | FOLDED | YES |
| **56** | **1304** | Outer membrane protein LolB | 21.1 | 17 - 193 aa | D | TRIMER | FOLDED | YES |
| **57** | **1304** | Outer membrane protein LolB | 21.1 | 17 - 193 aa | D | DIMER | FOLDED | YES |
| **58** | **1304** | Outer membrane protein LolB | 21.1 | 17 - 193 aa | D | MONOMER | FOLDED | YES |
| **59** | **1305** | Tetratricopeptide repeat family protein | 66.2 | 24 - 614 aa | A | MONOMER | INCONCLUSIVE | NO |
| **60** | **1397** | L-cystine ABC transporter, periplasmic L-cystine-binding protein | 28.7 | 17 - 274 aa | A | DIMER | FOLDED | NO |
| **61** | **1397** | L-cystine ABC transporter, periplasmic L-cystine-binding protein | 28.7 | 17 - 274 aa | A | MONOMER | FOLDED | NO |
| **62** | **1401** | Uncharacterized Protein | 17.0 | 21 - 159 aa | B | DIMER | FOLDED | NO |
| **63** | **1486** | Outer membrane protein assembly factor BamD | 29.0 | 19 - 267 aa | A + 5% glycerol | MONOMER | INCONCLUSIVE | NO |
| **64** | **1488** | IgA-specific serine endopeptidase | 131.8 | 30 - 1220 aa | D | MONOMER | INCONCLUSIVE | YES |
| **65** | **1520** | Aminodeoxychorismate lyase family protein | 34.5 | 22 - 331 aa | A | MONOMER | FOLDED | NO |
| **66** | **1525** | Neisserial Surface Protein A (NspA) | 16.6 | 20 - 174 aa | D | MONOMER | FOLDED | YES |
| **67** | **1525** | Neisserial Surface Protein A (NspA) | 18.3 | 1 - 174 aa | D | DIMER | FOLDED | YES |
| **68** | **1556** | Iron (III) ABC transporter, periplasmic Iron (III) binding protein | 35.8 | 23 - 331 aa | A | MONOMER | FOLDED | NO |
| **69** | **1567** | Putative putrescene ABC transporter, periplasmic putrescine-binding protein | 41.2 | 20 - 379 aa | A | DIMER | FOLDED | NO |
| **70** | **1567** | Putative putrescene ABC transporter, periplasmic putrescine-binding protein | 41.2 | 20 - 379 aa | A | MONOMER | FOLDED | NO |
| **71** | **1568** | LolA | 22.3 | 26 - 207 aa | A | MONOMER | FOLDED | NO |
| **72** | **1604** | ABC transporter, periplasmic substrate-binding protein | 33.3 | 20 - 304 aa | A | MONOMER | FOLDED | NO |
| **73** | **1640** | DSBA Thioredoxin | 25.2 | 18 232 aa | A | MONOMER | FOLDED | NO |
| **74** | **1714** | Hemolysin Secretion Activation ShlB/ Fhac/ HecB Family Protein | 67.1 | 32 - 595 aa | D | MONOMER | FOLDED | YES |
| **75** | **1758** | Type IV pilus biogenesis and competence protein PilQ | 80.8 | 25 - 777 aa | D | MONOMER | FOLDED | YES |
| **76** | **1758** | Type IV pilus biogenesis and competence protein PilQ | 59.9 | 25 - 580 aa | D | MONOMER | FOLDED | YES |
| **77** | **1812** | Factor H Binding Protein | 27.8 | 31 - 272 aa | A | MONOMER | FOLDED | NO |
| **78** | **1839** | Lipoprotein Mlp | 19.2 | 23 - 271 aa | A | MONOMER | FOLDED | NO |
| **79** | **1884** | Lipoprotein | 31.2 | 2 - 287 aa | A | MONOMER | FOLDED | NO |
| **80** | **1887** | Transglycosylase SLT domain protein | 67.7 | 27 - 616 aa | A + 5% glycerol | MONOMER | FOLDED | NO |
| **81** | **1887** | Transglycosylase SLT domain protein | 67.7 | 27 - 616 aa | A + 5% glycerol | DIMER | FOLDED | NO |
| **82** | **1887** | Transglycosylase SLT domain protein | 65.0 | 40 - 460 aa | D | MONOMER | FOLDED | YES |
| **83** | **1900** | Conserved Hypothetical Protein | 10.0 | 2 - 92 aa | A | MONOMER | INCONCLUSIVE | NO |
| **84** | **1926** | Fet A | 79.6 | 23 - 720 aa | D | DIMER | FOLDED | YES |
| **85** | **1926** | Fet A | 79.6 | 23 - 720 aa | D | MONOMER | FOLDED | YES |
| **86** | **1982** | PorB | 33.8 | 20 - 331 aa | D | MONOMER | FOLDED | YES |
| **87** | **2037** | Phospholipid binding domain protein (GNA2091) | 21.7 | 27 - 202 aa | A | MONOMER | FOLDED | NO |
| **88** | **2041** | Adhesin Complex protein | 12.3 | 22 - 124 aa | A | MONOMER | FOLDED | NO |
| **89** | **2074** | NHBA | 34.9 | 20 - 340 aa | D | MONOMER | FOLDED | YES |
| **90** | **2080** | Phospholipase Patain Family Protein | 31.3 | 29 - 300 aa | D | MONOMER | FOLDED | YES |
| **91** | **NMB1994** (From MC58 strain) | Neisseria Adhesin A | 31.2 | 26 - 310 aa | A | TRIMER | FOLDED | NO |

| ^¥^ All proteins were expressed from *Neisseria meningitidis* serogroup B H44/76 strain except NadA (NMB1994) which was obtained from the NmB strain MC58. |
| --- |
| ^§^ Buffer **A**: 25mM Hepes/NaOH pH 7.5, 150mM NaCl; Buffer **B**: 25mM Hepes/NaOH pH 7.0, 150mM NaCl; Buffer **C**: 25mM Tris/HCl pH 7.2, 300mM NaCl 1mM DTT 5% glycerol; Buffer **D**: 20 mM Tris/HCl pH 7.4, 150mM NaCl 0.1% LDAO |
